# Upcycling of sugar refining mud solid waste as a novel adsorbent for removing methylene blue and Congo red from wastewater†

**DOI:** 10.1039/d4ra01451k

**Published:** 2024-04-30

**Authors:** Aly Reda Aly, Abdel-Ghafar El-Demerdash, Wagih Sadik, Essam El Rafy, Tamer Shoeib

**Affiliations:** a Materials Science Department, Institute of Graduate Studies and Research, Alexandria University Alexandria Egypt; b Department of Chemistry, The American University in Cairo Egypt T.Shoeib@aucegypt.edu

## Abstract

The feasibility of utilizing the mud solid waste (MSW) produced during the carbonation process of sugar refining as a cost-effective and environmentally friendly alternative for the water removal of methylene blue (MB) and Congo red (CR), being highly utilized organic dyes representing cationic and anionic species, respectively is presented. Prior to its use, the MSW was dried at 110 °C for 24 h and sieved through a 100-mesh screen. The chief constituent of the MSW utilized was CaCO_3_, with a point of zero charge (PZC) found at pH 8.4 and 7.96 m^2^ g^−1^ total surface area. XRD and FTIR data indicate the presence of interactions between the dyes and the MSW surface, indicating effective adsorption. Different variables, such as initial dye concentration, MSW weight, solution pH, contact time, and temperature, were all examined to determine the optimal dye removal conditions. A central composite design (CCD) approach based on response surface methodology (RSM) modeling was utilized to identify statistically significant parameters for MB and CR adsorption capacities onto the MSW adsorbent. The removal equilibrium was typically reached in 120 minutes, with the greatest removal efficiency of CR taking place at pH 2 and 328 K, while the highest MB removal efficiency was obtained at pH 12 and 296 K. Kinetic studies suggest the adsorption of both dyes on the MSW follow pseudo-second-order rates, as evident through the high correlations obtained. Linearized and non-linearized Langmuir models showed strong correlations indicating maximum adsorption capacities of 86.6 and 72.3 mg g^−1^ for MB and CR, respectively. High regeneration and reusability potential of the MSW was demonstrated especially for the adsorption of CR, where the removal efficiency was nearly constant throughout five adsorption cycles, ranging from 93 to 91%, while the reduction in the removal for MB was much more significantly impacted, diminishing from 95 to 79% after the five cycles.

## Abstract 2

The feasibility of utilizing the mud solid waste produced during sugar refining as a cost-effective and environmentally friendly alternative for the removal of methylene blue and Congo red from wastewater is presented.

## Graphical abstract

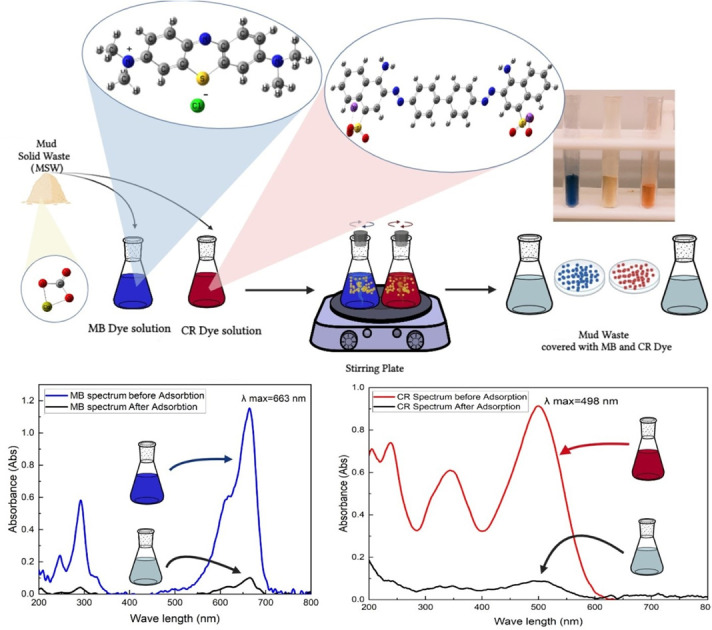

## 1. Introduction

Increased industrialization, population growth, and intensive agricultural practices have resulted in a significant rise in waste production, posing a critical global challenge for effective waste management.^1^ Dyeing, printing, paper, textile, and skin care industries utilize a plethora of dyes^2–4^ and their discharge into the hydrosphere. Due to their non-degradable characteristics, photo-stabilities, and affinities for oxidation, many dyes pose a threat to the ecosystem and human health.^5^ The toxic nature of dyes has prompted the exploration of various emerging materials for their remediation. For example, hydrogel polymers, metal oxides, composites, layered double hydroxides, and treated biosorbents are among the materials being studied for their efficient adsorption of dyes from water, a topic that has gathered significant recent attention and is the focus of this work.^6–10^

MB and CR were chosen as model dyes here as they are very highly utilized organic dyes, owing to their excellent color durability and water solubility in the dyeing of textiles, paper, and silk while representing cationic and anionic species, respectively.^11,12^ MB is a carcinogenic, teratogenic, and embryotoxic environmentally persistent cationic dye that poses significant health risks and environmental threats.^13^ At doses higher than 5 mg kg^−1^, it can trigger acute lethal serotonin toxicity in humans, affecting the respiratory, cardiovascular, genitourinary, and central nervous systems, as well as reducing sperm motility.^13^ In aquatic environments, MB negatively impacts chemical and biological oxygen demand and photosynthesis with clear detrimental effects.^14^ CR, on the other hand, is an anionic benzidine-based diazo dye that has been reported to negatively affect vision, respiration, and reproduction and cause allergic reactions in humans. CR is also known to produce benzidine as a primary mutagenic, cytotoxic, and carcinogenic metabolite which is in turn linked to hepatocarcinoma, splenic sarcoma, nuclear abnormalities, and chromosomal errors in mammalian cells as well as the induction of cancer of the bladder in humans and several cancers in animals.^15,16^ CR is also an environmental hazard hindering chlorophyll biosynthesis and green algae growth.^15,17^

Previous studies have explicitly focused on MB and CR, highlighting the effectiveness and potential of different adsorbents as remediation agents for these dyes.^8,10,18–21^ However, some common challenges related to producing adsorbents are high production and operational costs as well as the degradation and non-reusability of adsorbents.^22^ In this work, we attempt to address some of these problems by utilizing sugar refining MSW, an agro-industrial waste produced in significant amounts during the sugar refining process from sugar beets and poses adverse environmental effects, as a novel cost-effective and environmentally friendly alternative for removing these dyes from water. The sugar refining process entails clarification involving carbonation to remove coarse and colloidal impurities without sacrificing the sugar content, resulting in a clarified syrup with reduced color and turbidity.^23^ The clarification phase of sugar production typically involves an injection of limewater and carbon dioxide, which facilitate the removal, through precipitation or flocculation, of non-sugar components. The solid waste generated during this process initially assumes a fine-grained form before ultimately agglomerating during the sugar crystallization process.^24^ The annual global production of such MSW was estimated at 36.8 million tons in 2017.^25^ Globally, the MSW generated amounts to ∼3% by weight of the total local sugar production, posing considerable disposal challenges.^26^ A recent study estimated the transportation cost of MSW waste for its disposal to be around 9.5 USD km^−1^ Mg^−1^ while the actual cost of disposal was estimated at 11 USD km^−1^ Mg^−1^.^27^ A limited percentage of the MSW generated is used as soil fertilizer, with a larger percentage of being relegated to landfills for disposal. This causes considerable environmental risks due to the release of toxic compounds when rainwater filters through this mud solid waste^28^ and through greenhouse gas emissions associated with the disposal of such MSW in landfills, as well as their use in fertilizers.^29^

The utilization of such MSW in diverse applications, including the production of paper, cement bricks, plastic fillers, biodiesel, and in improving glass powder performance has been the subject of recent reports.^29–32^ These reports provide compelling evidence for the versatility and value of this MSW as a sustainable resource across multiple industries. Recent research efforts have also suggested that various bio-wastes may be used as sustainable and cost-effective alternatives to synthetic materials for water removal of dyes.^33–36^ In fact, adsorption has emerged as a preferred method for removing carcinogenic dyes and heavy metals from industrial wastewater among traditional treatment techniques like electrocoagulation, coagulation–flocculation, membrane filtration, chemical precipitation, and ion exchange. This is partly due to the relatively straightforward design, low operating cost, and excellent efficiency of adsorption techniques.^37,38^ Many studies have identified CaCO_3_ as a safe and environmentally promising material for removing dyes and heavy metals with acceptable efficiency.^39–46^ Previous studies have shown CaCO_3_ as a chief constituent of MSW providing additional motivation for exploring it here as a novel dye adsorbent material.^47^

Finally, this work presents data to assess the feasibility of MSW as an eco-friendly adsorbent for the water removal of CR and MB under several conditions to reduce their environmental impact and promote sustainability. The impact of several factors on the adsorption efficiency of the MSW, including contact time, solution pH, temperature, initial dye concentration, and amount of MSW adsorbent, were assessed. The adsorption kinetics, thermodynamics, and isotherms models are discussed.

## 2. Experimental procedures

### 2.1 Materials

The MSW produced during the process of sugar refining and employed in this work was obtained from The Nile Sugar Factory (Alexandria, Egypt). MB, CR, sodium hydroxide pellets, potassium nitrate, nitric acid, and hydrochloric acid were all obtained from Sigma-Aldrich. Two dye stock solutions of 1000 ppm were prepared in distilled water. Experimental solutions of 10, 25, 50, 75, 100, 125, 150, 175, 200, 225 and 250 ppm were obtained through serial dilutions.

### 2.2 MSW characterization

The MSW was oven dried at 110 ± 5 °C for 24 hours and then sieved through a 100-mesh screen. The phase composition of the MSW before and after MB and CR adsorption were examined by X-ray diffraction (XRD) analysis employing a D8 Discover Bruker instrument employing Cu Kα radiation (*λ* = 0.154 nm) set at 40 kV and 40 mA within the 2*θ* range of 20 to 80° with a 0.02° step size. A JEOL JSM 6360 LA scanning electron microscope equipped with energy-dispersive X-ray spectroscopy was employed to observe the morphology of the MSW samples before and after dyes adsorption. Fourier transformation infrared (FTIR) spectra for the MSW before and after MB and CR adsorption were obtained using a Nicolet 380 FTIR spectrometer. The MSW powder was blended with KBr and pressed to form pellets. The measurement was performed at room temperature, covering a spectral range of 400 to 4000 cm^−1^ with a resolution of ±1 cm^−1^. The gas adsorption–desorption isotherms, surface area, and porosity of the MSW were all determined using data gathered from a Micromeritics ASAP 2020 device. The powder addition method was employed to identify the point of zero charge (PZC) of the MSW.^48,49^ In separate 100 ml conical flasks, 50 ml of 0.1 M KNO_3_ solution was transferred, and the initial pH values were modified within the 2–12 range by adding either 0.1 N HNO_3_ or 0.1 N NaOH. To each conical flask, 2 g L^−1^ of MSW was added and stirred for 24 h, and the final pH values of the supernatant were determined.

### 2.3 Adsorption tests

Adsorption tests were conducted in a batch process for both dyes. The effects of contact time, initial dye concentration, and temperature were described in detail and listed in Text S1 as well as Table S1 of the ESI.† After centrifugation at 5000 rpm for 5 min to partition the dye solutions from the adsorbent, the final concentrations of dyes in the supernatant were determined using a Jenway 7415 UV-visible spectrophotometer. Equations eqn S(1)–S(15)† utilized in the study of adsorption test, kinetic modeling, isotherm modeling and thermodynamics in detail and listed in the ESI.†

### 2.4 Optimization of MB and CR adsorption onto the MSW

The optimum conditions for the adsorption process were assessed using the RSM in conjunction with CCD.^50^ This approach was shown to be successful in adsorption process optimization while providing a refined perspective for developing predictive designs.^50,51^ In the ESI,† the study provides detailed descriptions of the experimental conditions and equations (eqn S(16) and S(17)) utilized, as presented in Text S2 and Table S2.†

## 3. Results and discussion

### 3.1 XRD and FTIR analysis

**Fig. 1:**
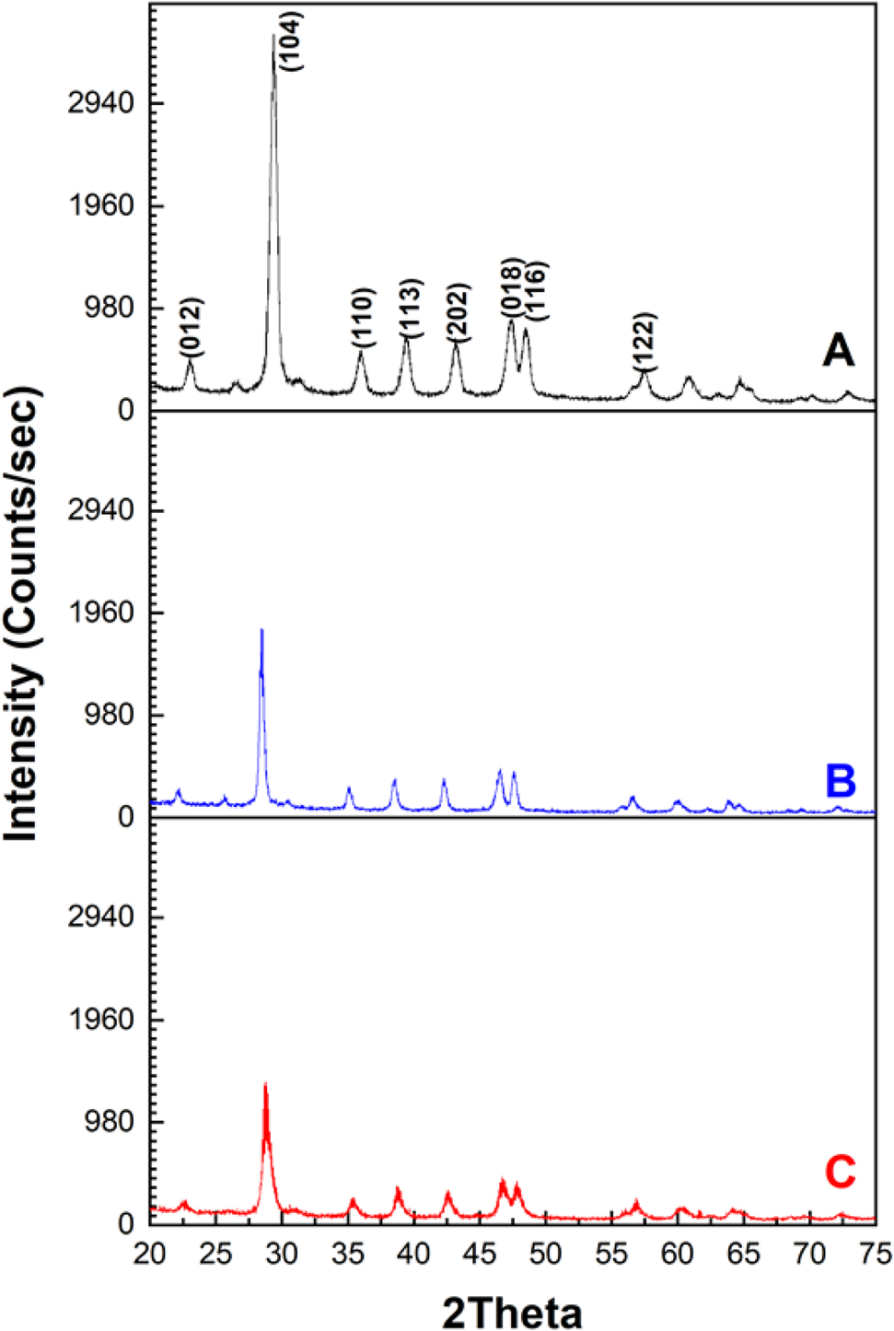
: XRD patterns of MSW before (Panel A) and after MB (Panel B) or CR (Panel C) adsorption.

The phase crystallography of the MSW before and after MB and CR adsorption is shown in Fig. 1. The XRD diffraction pattern of the MSW was found to match that of rhombohedral CaCO_3_. The main peaks, in the order of descending intensities, are at 2*θ* values of 29.50°, 47.53°, 48.56°, 39.54°, 43.31°, 36.10°, 23.15°, and 57.57° with miller indices of planes (104), (018), (116), (113), (202), (110), (012), and (122), respectively. This data indicates that the majority of the MSW sample is composed of calcite calcium carbonate, CaCO_3_.^52,53^ The average crystallite size of the MSW employed was determined through the Debye–Scherer equation using the values 1.54 Å and 0.9 for the X-ray wavelength and the dimensionless shape factor *K* respectively and found to be 23.6 nm.^54^ A comparison of XRD data of either MB or CR adsorbed on the MSW shows a reduction in the intensity of all peaks due to the interaction between the dyes and the MSW surface, indicating effective adsorption.

In line with previous reports, the reduction in the intensities of the XRD peaks of the MSW after the adsorption of dyes observed here, is most likely due to structural changes in the MSW due the formation of new chemical species providing indirect evidence of effective adsorption.^55–57^

**Fig. 2:**
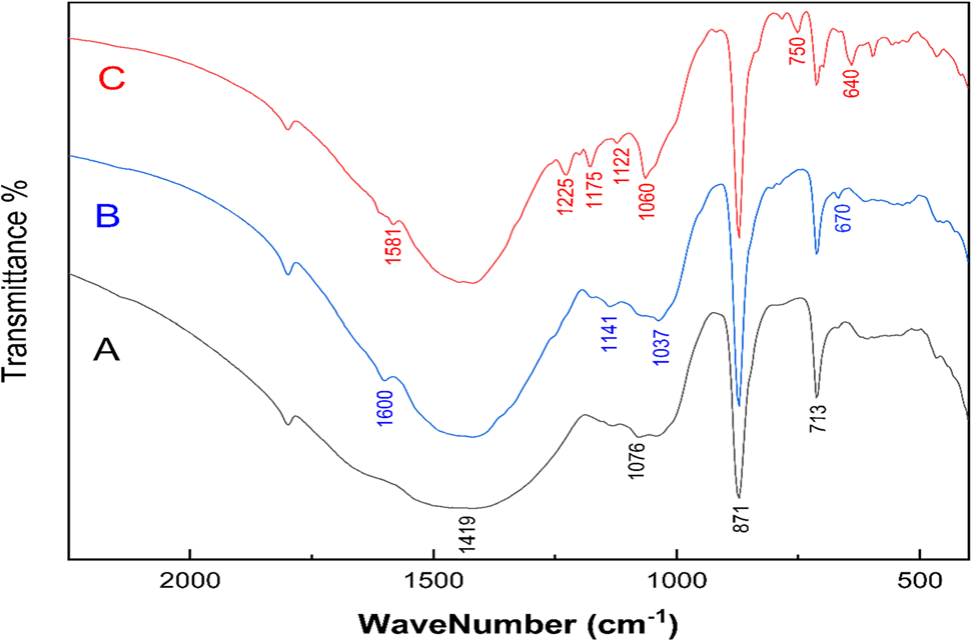
FTIR spectrums of MSW before and after MB or CR adsorption. (Trace A) pristine MSW, (Trace B) MSW after MB adsorption, and (Trace C) MSW after CR adsorption.

The FTIR spectrum of the MSW before and after MB or CR adsorption is shown in Fig. 2. Observed bands at 713, 871, 1076, and 1419 cm^−1^ are assigned to the in-plane bending, out of plane bending, symmetric stretching of the C–O bond, and asymmetric stretching of C–O bond of the carbonate group, CO_3_^2−^, respectively.^26,58^ The FTIR spectrum of the MSW is consistent with the typical vibrations of calcite calcium carbonate, as previously reported.^52,59^ The adsorption of MB on the MSW results in the appearance of low intensity bands at 1600 cm^−1^, which are most likely due to C=N and C=C stretching vibrations, while the band at 1141 cm^−1^ is attributed to stretching vibrations of C–N, the bands at 1037 and 670 cm^−1^ correspond to asymmetric and symmetric vibrations of C–S–C, respectively.^60^ The interaction of CR with the MSW is characterized by the emergence of new bands at 1581, 1225, 1060, and 750 that are assigned to C=C stretching, C–N stretching, S=O stretching, and N–H wagging, respectively. The bands at 1175, 1122, and 640 are assigned to asymmetric and symmetric stretching vibrations of SO_3_.^61^ The appearance of new bands in the FTIR spectrum suggests the successful adsorption of MB and CR onto the MSW.

### 3.2 Morphology and elemental analysis

The SEM image in Fig. S1 in the ESI† shows the surface morphology of the MSW before and after dyes adsorption. As shown in Fig. S1a,† MSW has the tendency to agglomerate and display the existence of particle clusters, indicative of the agglomeration that is prevalent within the MSW powder. The morphological analysis of MSW powder demonstrates the presence of an irregular crystal structure. Following the dyes adsorption on SMW, differences in the surface morphology of MSW were observed. A considerable quantity of microsphere particles was settled on the surface of MSW thereby indicating the successful adsorption of CR and MB as shown in Fig. S1b and c in the ESI.† The elemental composition of the MSW in Fig. S2 and Table S3 in the ESI† show that calcium, oxygen, and carbon are the major constituents of the MSW making up 93.14% of its total composition. This aligns with the XRD and FTIR data suggesting that CaCO_3_ is the predominant species in the MSW.

The surface morphology of CMW before and after dyes adsorption was analyzed by SEM.

### 3.3 BET adsorption analysis and PZC of the MSW

The BET model displayed in Fig. S3 in the ESI† is classified as a Type II isotherm^62^ that demonstrates significant interaction involving macroporous adsorbents. The total surface area was calculated to be about 7.96 m^2^ g^−1^, with a pore volume of 0.0271 cm^3^ g^−1^. Fig. S3 and S4 in the ESI† depict the obtained pore size distribution, which ranged from 2.7 to 145.8 nm in pore diameter, suggesting that certain mesopores coexist with macro pores. The PZC is directly related to the pH value at which the surface of the adsorbent exhibits net electrical neutrality. At pH levels lower than the PZC, anions will be more desirable for adsorption, whereas adsorption of cations will be more amenable at pH values higher than the PZC. A PZC value of 8.4 was obtained for the MSW as shown in Fig. S5 in the ESI† which is in line with those previously reported for calcite.^63–65^ At pH values below 8.4, the MSW surface is thus largely occupied by positive species such as Ca^+2^, CaOH^+^, and CaHCO_3_^+^ that interact more effectively with anionic while at pH higher than 8.4 negative species such as HCO_3_^−^ and CO_3_^−2^ dominate the MSW surface enabling effective interaction with cationic dyes.^63,66,67^

### 3.4 Adsorption analysis

#### 3.4.1 Contact time and kinetics

**Fig. 3:**
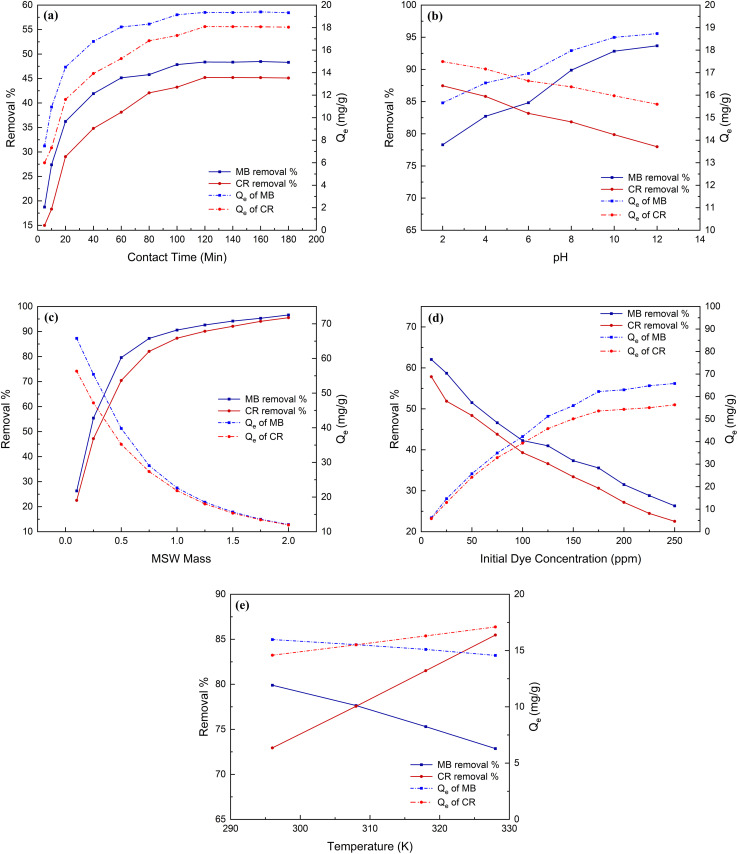
MB and CR dyes removal % and adsorption capacity affected by contact time (Panel a), pH (Panel b), MSW weight (Panel c), initial dye concentrations (Panel d), and temperature (Panel e).

Fig. 3 (Panel a) shows the percent removal of 100 ppm of either MB or CR mixed with 0.25 g of the MSW in the contact time range of 5–180 min. This figure shows an increased percent removal for MB and CR from 18.7 to 48.3 and from 15.0 to 45.1, respectively, in the range of 5 to 120 min. The removal percentages for both MB and CR were relatively constant past 120 min contact time, suggesting equilibrium being reached. The adsorption capacities of MB and CR were also observed to increase from 7.5 to 19.4 mg g^−1^ and from 6.0 to 18.1 mg g^−1^, respectively, with increasing contact time from 5 min to up until equilibrium at the optimum contact time of 120 min which is consistent with other studies employing eggshell as an adsorbent in which calcium carbonate was the main constituent.^68,69^ Furthermore, the utilization of the MSW to remove MB and CR demonstrates a considerably shorter equilibrium time compared to chitosan–graphene oxide and biochar, which necessitated approximately 48 and 60 h respectively.^70,71^

**Fig. 4:**
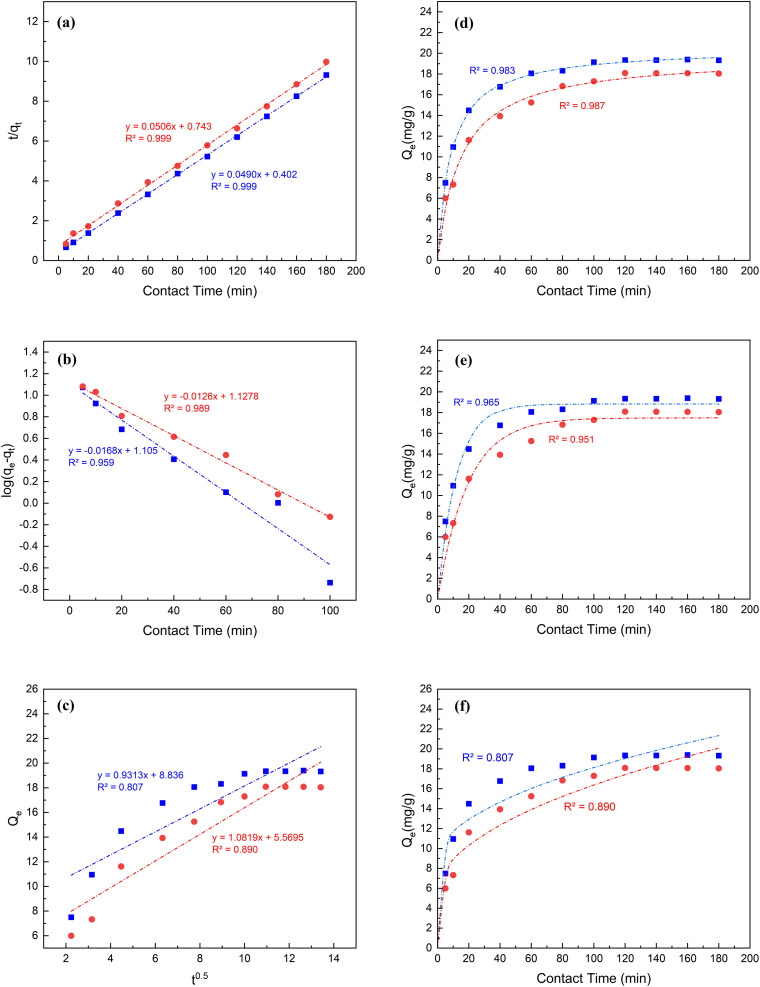
Panels a–c are the linearized forms of the pseudo second-order, pseudo first-order, and intra-particle diffusion kinetics models respectively. Panels d–f are the non-linearized forms of second-order pseudo, first-order Pseudo, and intra-particle diffusion kinetics models, respectively all for the adsorption of MB and CR on MSW represented by squares and circles, respectively.

A detailed description of constructing linear and nonlinear kinetics models is described in Text S3 in the ESI.† The data presented in Fig. 4 and Table 1 suggest that pseudo-second-order models most accurately characterized the kinetics of the adsorption for both MB and CR. The hypothesis of linearized and non-linearized forms of pseudo-second order are consistent with other studies^72,73^ and are endorsed by the strong correlation values of 0.999 and 0.983 for the adsorption of MB as well as 0.999 and 0.987 for the adsorption of CR, respectively onto the MSW. The adsorption capacities *Q*_e2_ of the MSW for MB and CR dyes are 20.4 and 19.8 mg g^−1^, for both linearized and non-linearized forms being in line with experimental values of 19.4 and 18.1 mg g^−1^ respectively.

Parameters of kinetic models, isotherm models, and thermodynamics of the adsorption of dyes using the MSW

**Table:** 

Adsorption kinetic parameters	Kinetic models	Parameters	Values			
			MB		CR	
			Linear	Non-linear	Linear	Non-linear
	Pseudo-first order	*K*_1_	0.0386	0.0837	0.0290	0.052
		*Q*_e1_	12.735	18.8008	13.422	17.490
		*R* ^2^	0.959	0.965	0.989	0.951
	Pseudo-second order	*K*_2_	0.006	0.006	0.003	0.003
		*Q*_e2_	20.404	20.515	19.845	19.749
		*R* ^2^	0.999	0.983	0.999	0.987
	Intra-particle diffusion	Kid	0.931	0.931	1.082	1.082
		*C*	8.840	8.836	5.569	5.569
		*R* ^2^	0.807	0.807	0.890	0.890

**Table:** 

Adsorption isotherm parameters	Isotherm models	Parameters	Values			
			MB		CR	
			Linear	Non-linear	Linear	Non-linear
	Langmuir form	*B*	0.019	0.017	0.020	0.019
		*Q*_max_	86.580	89.177	72.309	72.968
		*R* ^2^	0.993	0.994	0.996	0.996
		*R*_L_	0.176	0.188	0.166	0.167
	Freundlich form	*K*_f_	16.219	6.021	12.526	5.909
		*n*	1.647	2.102	1.677	2.245
		*R* ^2^	0.975	0.966	0.963	0.953

**Table:** 

Thermodynamic parameters	Temperature (K)	Parameters	Values	
			MB	CR
	296 K	Δ*G*° (J mol^−1^)	−3436.70	−2427.59
	308 K		−3198.33	−3216.04
	318 K		−2959.96	−4004.49
	328 K		−2721.60	−4792.94
		Δ*H*° (kJ mol^−1^)	−10.54	21.08
		Δ*S*° (J K^−1^ mol)	−23.84	78.84

#### 3.4.2 Effect of pH

The effect of pH on MB and CR adsorption onto the MSW is presented in Fig. 3 (Panel b). The elevation of the solution pH from 2 to 12 resulted in increasing the percent removal and *Q*_e_ of MB from 78.3 to 93.7 and from 15.7 to 18.7 mg g^−1^ respectively and the reduction of the percent removal and *Q*_e_ of CR from 87.5 to 78.0 and from 17.5 to 15.6 mg g^−1^ respectively. This is most likely due to increased electrostatic attraction to the MB cationic dye and the increased electrostatic repulsion to the CR anionic dye with the increased concentration of hydroxyl ions.^73^

#### 3.4.3 MSW mass

The mass of the MSW was a parameter examined in our efforts to realize the most efficient conditions for removing the two dyes under study. Fig. 3 (Panel c) shows a trend of increased removal of both dyes examined at a concentration of 250 pm with increased MSW weight, where 1 g of MSW removes over 85% of both dyes while 1.5 g of MSW is the amount at which the plateau in the percent removal of both dyes starts to be observed.

#### 3.4.4 Initial dye concentrations and isotherm models

**Fig. 5:**
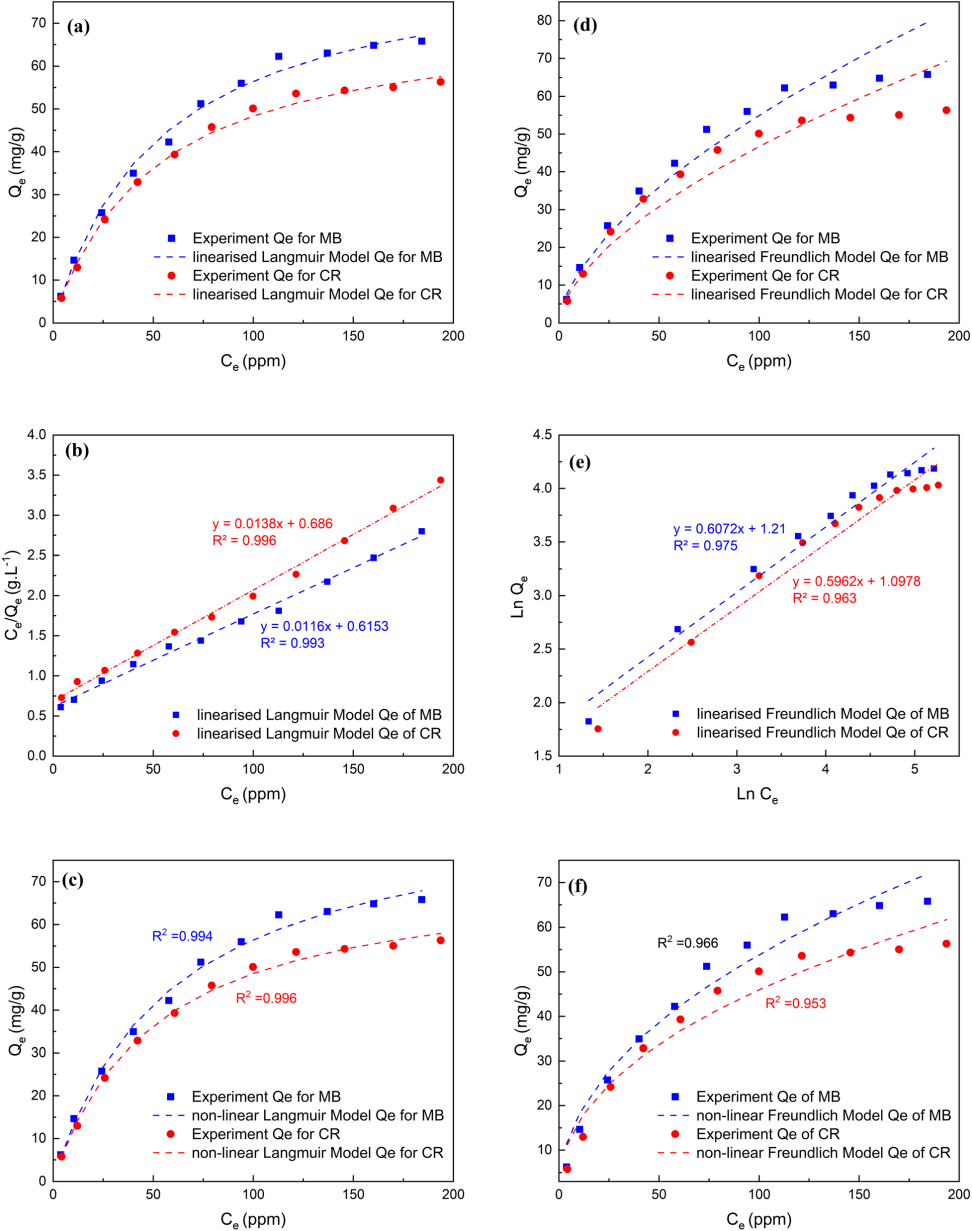
Panels a and b are for linearized, Panel c is for non-linearized Langmuir isotherm Mobels, while Panels d and e are for linearized, Panel f is for non-linearized Freundlich model isotherm models all for the adsorption of MB and CR on MSW represented by squares and circles, respectively.

Fig. 3 (Panel d) demonstrates the impact of initial MB and CR dye concentration on their removal. The removal percentage of MB and CR of different initial dyes mixed with 0.10 g of MSW decreased from 62.1 to 26.3 and from 57.9 to 22.5, respectively. While the adsorption capacities of MB and CR by the MSW increased from 6.2 to 65.8 mg g^−1^ and from 5.8 to 56.3 mg g^−1^, respectively. According to these results, the removal of MB and CR by the MSW is related to the initial dye concentration that provides the driving forces needed to overcome the resistances of dyes to migrate from the solutions to the MSW surface.^69^ As shown in Fig. 5 and listed in Table 1 the adsorption isotherm was modeled using linearized and non-linearized Langmuir and Freundlich equations, with correlations of 0.994 and 0.966 for MB and 0.996 and 0.953 for CR respectively. While both models exhibit good fitting agreement with the experimentally obtained adsorption isotherms, the Langmuir model shows higher correlation coefficient values implying the adsorption to be best described as monolayer processes with Langmuir *Q*_max_ values of 86.6 and 72.3 mg g^−1^ for MB and CR respectively. The observed superior adsorption performance of the MSW for MB can be attributed to the solution having a pH of 9.4 being higher than the PZC of 8.4 which increases the electrostatic attraction of positive MB ions onto the surface of the MSW.^67,74^ However, the *R*_L_ values of 0.176 and 0.166 for MB and CR respectively suggest the adsorption process is favorable.^75^

Values of *Q*_max_ reported for MB and CR removal from water in previous literature for other adsorbents derived from treated and untreated agro-industrial waste are presented in Table 2. By comparing these values to those obtained in this study, it is evident that for both MB and CR removal the untreated MSW employed here is superior to most of the other untreated agro-industrial derived adsorbents. In these previous studies, the capacities ranged from 19.7 to 97.1 mg g^−1^ and 18.2 to 49.5 mg g^−1^ for MB and CR respectively. However, the performance of untreated MSW relative to treated agro-industrial derived adsorbents is less impressive, outperforming only about half of the reported treated agro-industrial derived adsorbents.

**Table:** Summary of several recent studies on MB and CR removal using treated and untreated agro-industrial wastes

Adsorbent	*Q*_max_ mg g^−1^		Reference
	MB	CR	
Untreated MSW	86.6	72.3	This study
Untreated egg waste matrix	94.9	49.5	88
Untreated orange peel waste	18.6	14.0	89
Untreated banana peel waste	20.8	18.2	89
Untreated potato peels waste	33.8	—	90
Untreated pineapple peels waste	97.1	—	91
Untreated dragon fruit peels waste	62.6	—	92
Untreated olive stone	44.5	—	93
Untreated walnut shell powder	—	18.5	94
Treated banana peels waste	19.7	—	95
Treated *U. fasciata* waste	45.9	31.0	96
Treated *S. dentifolium* waste	65.8	28.2	96
Treated ashitaba waste	381.9	632.1	97
Treated walnut shell	400.1	442.6	97
Treated streptomyces fradiae biomass	59.6	46.6	98
Treated einkorn husk	151.5	—	99
Treated walnut shell	—	40	94
Treated sugarcane waste	—	102.3	100
Treated rice straw composite	—	34.2	101
Treated *Cornulaca-monacantha* stem	—	78.2	102
Treated walnut shell	—	44.4	103

#### 3.4.5 Temperature influence and thermodynamics

The dependence of dye removal and the adsorption capacity upon temperature variation from 296 to 328 K has been studied and is shown in Fig. 3 (Panel e). The results indicate that temperature elevation from 296 to 328 K results in an increased percentage of CR removal from 72.9 to 85.5% and increased *Q*_e_, the amount of CR adsorbed onto the MSW, from 14.6 to 17.1 mg g^−1^. This is most likely due to the increased diffusion of CR molecules from the bulk solution toward the surface of the MSW at higher temperatures.^76^ Inversely, the MB removal percentage declined from 79.9 to 72.9 while the associated *Q*_e_ decreased from 16.0 to 14.6 mg g^−1^ with increasing temperature from 296 to 328 K. This is most likely due to increased solubility of the MB dye with increasing temperature thus reducing the adsorption onto the MSW surface.^77,78^ Thermodynamic parameters for the adsorption of CR and MB on the MSW are shown in Table 1 and Fig. S6 in the ESI.† From these parameters it is clear to see that the CR adsorption process is endothermic while that of MB adsorption is exothermic.

#### 3.4.6 Proposed adsorption mechanisms

**Fig. 6:**
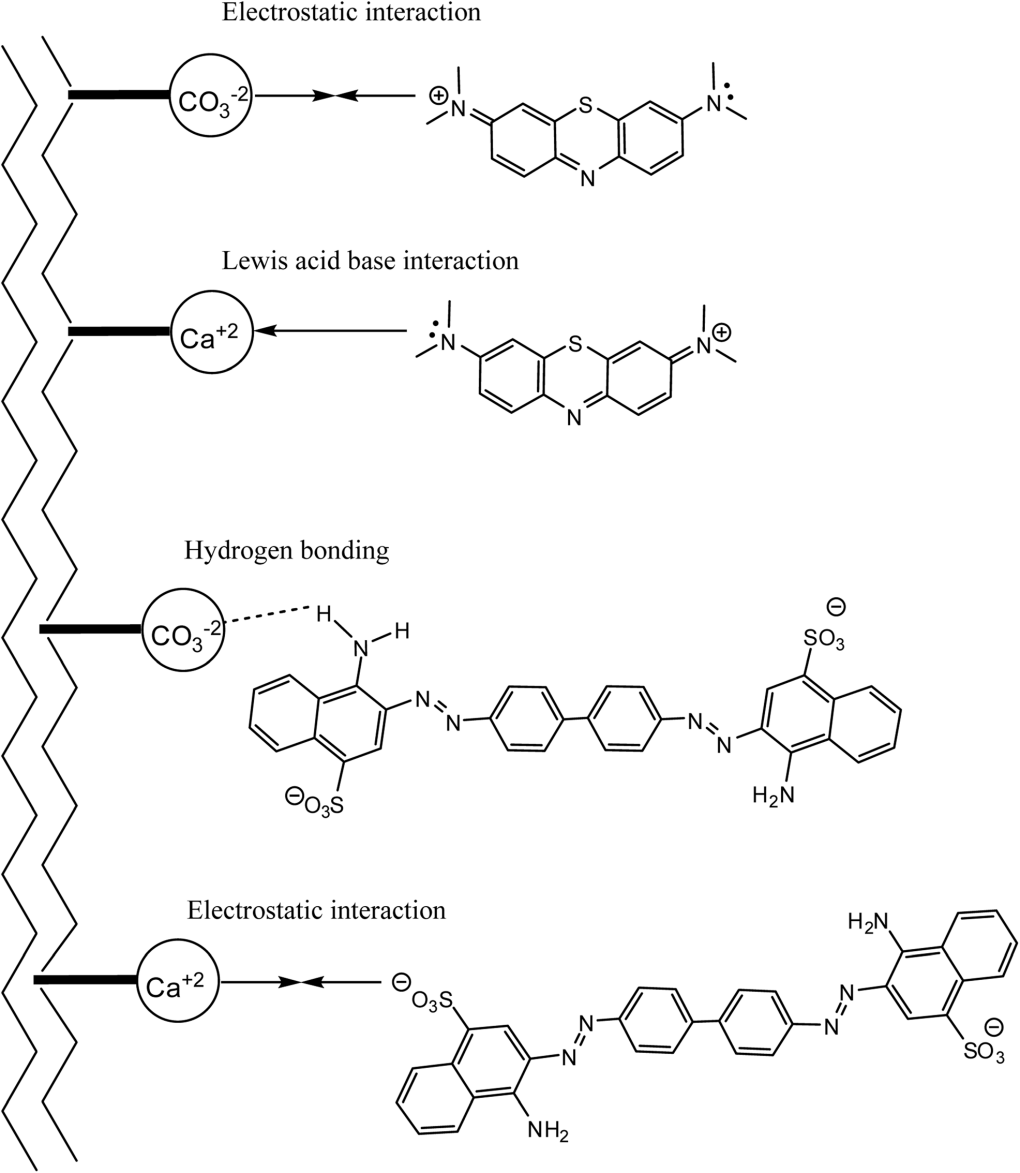
Proposed mechanisms for the adsorption of MB and CR onto the MSW. The top two interactions illustrate the proposed adsorption mechanisms of MB while the bottom two illustrate the proposed adsorption mechanisms of CR onto the MSW respectively.

Fig. 6 proposes that MB most likely adsorbs onto the MSW *via* two strong interactions, electrostatic and Lewis acid-base.^43,79,80^ The latter of these interactions would not be favorable in acidic media which is in line with the observation of decreased MB adsorption of MB onto the MSW at lower pH values. In the cases of CR, it is proposed that adsorption onto the MSW most likely takes place through electrostatic interactions as well as the weaker physical interaction being hydrogen bonding, this is consistent with the overall lower value of *Q*_max_ for CR relative to that obtained for MB.^73,81,82^

### 3.5 ANOVA-validated RSM-CCD optimization and visualization

To optimize the adsorption parameters of MB and CR removal capacities using the MSW adsorbent RSM-CCD modeling was utilized. Table S4 in the ESI† lists the complete design matrix that consists of a variety of combinations of the four variables, along with the experimental and predicted amounts of *Q*_e_ for MB and CR. The various factors in the study are accompanied by their corresponding regression coefficients, Student's *t*-test values, *p*-values, and standard errors with a confidence level of 95% (*p* < 0.05) in Table S5 in the ESI.† A higher level of significance is generally associated with a larger *t*-value and a smaller *p*-value for a coefficient term.^83^

The coefficient with one variable represents the effect of an individual variable, whereas the coefficients with a pair of variables and with a second-order term represent the correlation between two variables and the quadratic effect, respectively. Accordingly, the initial dye concentration (*X*_1_), the MSW weight (*X*_2_) and its quadratic term (*X*_2_^2^), temperature (*X*_3_), as well as and the correlation term between (*X*_1_) and (*X*_2_) were significant in the case of the CR dye, whereas for the MB dye, the significant parameters were the initial dye concentration (*X*_1_), the MSW weight (*X*_2_) and its quadratic term (*X*_2_^2^), pH (*X*_4_), as well as the correlation term between (*X*_1_) and (*X*_2_).

The quadratic parameters (*X*_1_^2^), (*X*_3_^2^) for CR, and (*X*_1_^2^), (*X*_4_^2^) for MB were less significant than their respective linear ones. All two-way interactions showed minimal effects and were statistically insignificant except for interaction between (*X*_1_) and (*X*_2_) in both CR and MB models, as shown in Fig. S7 in the ESI.† The uncoded quadratic empirical equations for the adsorption capacities of CR and MB in terms of independent and dependent variables are represented by eqn (18) and (19),† respectively while the corresponding equations showing only significant dependent variables by using the backward elimination approach, are represented by eqn (20) and (21) all in the ESI.†

**Fig. 7:**
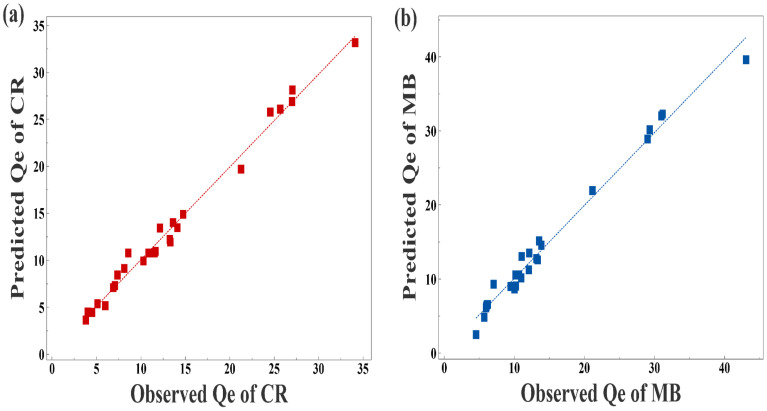
Panel a is predicted *Q*_e_*vs.* observed *Q*_e_ of CR Model. Panel b predicted *Q*_e_*vs.* observed *Q*_e_ of MB Model.

**Fig. 8:**
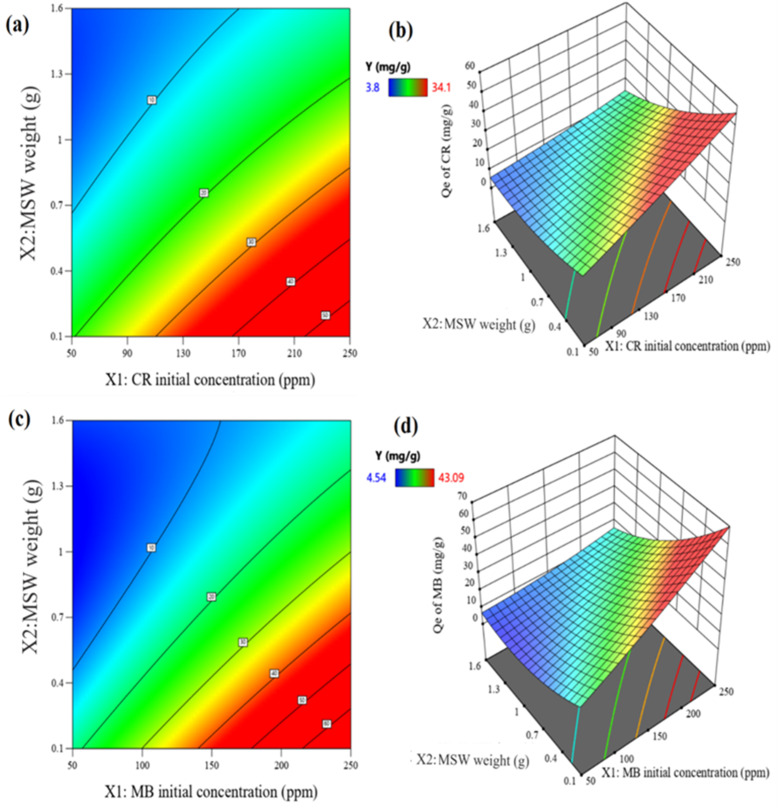
Panel a is contour plot showing the impact of both *X*_1_ with *X*_2_ on *Q*_e_ of CR at pH = 2 and 328 K. Panel b is a 3D surface plot representing the impact both *X*_1_ with *X*_2_ on *Q*_e_ of CR at pH = 2 and 328 K. Panel c is a contour plot showing the impact of both *X*_1_ with *X*_2_ on *Q*_e_ of MB at pH = 12 and 296 K. Panel d is a 3D surface representing the impact both *X*_1_ with *X*_2_ on *Q*_e_ of MB at pH = 12 and 296 K.

The indications of synergistic and opposing influences among the variables are demonstrated by the signs of the regression coefficients. For eqn (3) and (4),† the initial dye concentrations (*X*_1_) and the quadric terms of the MSW weight (*X*_2_^2^) for both dyes as well as the temperature (*X*_3_) in the case of CR and pH (*X*_4_) in the case of MB all have significant positive effects on the adsorption capacities. On the other hand, the MSW weights (*X*_2_) and the interaction terms between (*X*_1_) and (*X*_2_) all have significant adverse impacts, as shown in Fig. S7 in the ESI.† Using a variance of analysis approach (ANOVA), the suitability of mathematical models for CR and MB was evaluated. The statistical significance of each variable was assessed by comparing its distribution of variance (*F*) with the respective probability. The is determined by large values of Fisher's test coefficients (*F* value) and small values of probability (*p* values) typically indicate the statistical significance of a regression model.^84^ A higher computed *F* value than the Table value of *F* in a particular number of degrees of freedom indicates the significance of the model and its capacity to predict experimental results.^85^ According to the ANOVA analyses listed in Table S6 in ESI,† the regression equations produced *F* values that were larger than the table values of *F* suggesting that the mathematical models adopted were suitable. The capacity to make accurate estimates of *Q*_e_ is also confirmed by high correlation coefficients *R*^2^ and adj-*R*^2^, which were 0.988 and 0.978 for CR as well as 0.984 and 0.970 for MB, respectively. The good fit between predicted and observed adsorption capacities are represented in Table S6 in ESI† and Fig. 7. Three-dimensional surface graphs and contour plots facilitate the visualization of statistical regression equations and highlight the optimum values of independent variables for higher response. It is noted that the highest *Q*_e_ values for CR and MB are obtained at an initial concentration of 250 ppm on 0.1 g of MSW while the lowest *Q*_e_ values are obtained at an initial concentration of 50 ppm with 2.1 g of MSW as represented in Fig. 8.

### 3.6 Regeneration and reusability of MSW

To assess the feasibility of regenerating and reusing the MSW, we employed 0.25 g of MSW at dye concentrations of 100 ppm and pH = 9.4 at room temperature, and after each adsorption cycle the MSW was rinsed several times with distilled water and then was dried at 450 °C for 2 hours in a muffle furnace to desorb the dyes.^86,87^ Fig. S8† shows the removal percentages of MB and CR by the regenerated MSW for five adsorption cycles. This figure shows the high regeneration and reusability potential, especially for the adsorption of CR, where the removal efficiency was nearly constant throughout the five cycles, ranging from 93 to 91%, while the reduction in the removal for MB was much more significant diminishing from 95 to 79% after the five cycles.

## 4. Conclusion

This study shows the feasibility of utilizing the mud solid waste produced during the carbonation process of sugar refining as a cost-effective and environmentally friendly alternative for the water removal of methylene blue and Congo red from aqueous solutions under various conditions. The chief constituent of the MSW was shown to be CaCO_3_ with a PZC at pH 8.4. The study has shown the optimal contact time for both dyes is 120 min and the efficacy of removing MB and CR increased with an increase in the MSW weight. Meanwhile, CR was more effectively removed in acidic media and higher temperatures, while an alkaline medium and room temperature were most effective for MB dye removal. The kinetic analysis demonstrates that pseudo-second-order models best describe the adsorption of both dyes with the maximum adsorption capacities estimated to be 86.6 and 72.3 mg g^−1^ for MB and CR respectively according to the Langmuir isotherm model. The adsorption of both dyes on the MSW was shown to be spontaneous and favorable.

Application of this eco-friendly approach for the utilization of MSW on an industrial scale for the removal of dyes and other contaminants such as heavy metals, and pharmaceuticals from water may warrant future investigation.

## Conflicts of interest

There are no conflicts to declare.

## Supplementary Material

RA-014-D4RA01451K-s001

RA-014-D4RA01451K-s002
