# Supplementary material for: Upcycling of sugar refining mud solid waste as a novel adsorbent for removing methylene blue and Congo red from wastewater
Source: RSC Adv. 2024 Apr 30;14(19):13505–20. doi: 10.1039/d4ra01451k (PMC11060308; doi:10.1039/d4ra01451k)
Supplement: RA-014-D4RA01451K-s001 [file RA-014-D4RA01451K-s001.pdf]

Electronic Supplementary Material (ESI) for RSC Advances.
This journal is © The Royal Society of Chemistry 2024

Supplementary material

Upcycling of Sugar Refining Mud Solid Waste as a Novel Adsorbent for Removing Methylene Blue and Congo Red from Wastewater.

Aly Reda Aly[a,b], Abdel-Ghafar El-Demerdash[a], Wagih Sadik[a], Essam El Rafy[a], Tamer Shoeib[b*]

a) Materials Science Department, Institute of Graduate Studies and Research, Alexandria University, Alexandria Egypt.
b) Department of Chemistry, The American University in Cairo, Egypt.

Corresponding author: Tamer Shoeib (T.Shoeib@aucegypt.edu)

Summary
Number of texts: 3
Number of tables: 6
Number of figures: 8
Number of equations:21

Table of Contents

Index	Captions	Page
**Text S1**	Experimental conditions of MB and CR adsorption tests with studying kinetic modeling, isotherm models, and thermodynamic parameters.	S1-S4
**Text S2**	Experimental conditions for the Optimization of MB and CR adsorption capacities onto the MSW via CCD and RSM	S4-S5
**Text S3**	A detailed description of constructing linear and nonlinear kinetics models.	S5
**Table S1**	Parameters and Experimental conditions of MB and CR adsorption tests	S4
**Table S2**	Coded levels of the independent parameters used in the RSM.	S5
**Table S3**	Elemental map analysis of the MSW	S15
**Table S4**	Observed and predicted adsorption capacities of the CCD model for the CR and MB adsorption on the MSW.	S16
**Table S5**	Predicted regression coefficients with SE, T-values, and P-values.	S17
**Table S6**	ANOVA of CR and MB dye adsorption capacities	S18
**Figure S1**	SEM image for the morphology of the MSW	S7
**Figure S2**	EDX spectra of elemental map analysis for the MSW	S8
**Figure S3**	BET isotherm type II significant interaction involving macroporous adsorbent CMSW.	S9
**Figure S4**	dispersion of the BET isotherm for pore size for the MSW.	S10
**Figure S5**	Point of zero charge (PZC) for the MSW from plot of Δ pH against initial pH.	S11
**Figure S6**	Thermodynamics for the adsorption of MB or CR on the MSW.	S12
**Figure S7**	Significant and insignificant regression coefficients of CR model (a) and MB Model (b).	S13
**Figure S8**	Figure S8: The removal percentage of MB and CR through adsorption onto MSW after five regeneration cycles.	S14
**Eq. S18-S21**	Response Surface Methodology Equations	S19
**References**		S20

1. Experimental procedures

Text S1: Experimental conditions of MB and CR adsorption tests with studying kinetic modeling, isotherm models, and thermodynamic parameters.

Text S1.1 Effect of contact time and kinetic modeling

The adsorption amount was measured against time to determine the adsorption kinetics of CR and MB removal processes. The equations eq1s and eq2s, as shown below in (SI) were applied to represent the amount of the adsorbed dyes at the equilibrium, Q_e (mg/g), and the percentage of removal efficiency R% by the MSW, respectively [1].

$$Q_e = \frac{(C_i - C_f)}{m} \times V \qquad \textbf{Eq. S1}$$

$$\mathbf{R\%} = \frac{(C_i - C_f)}{C_i} \times 100 \qquad \textbf{Eq. S2}$$

where C_i, C_f, m, and V are the initial dye concentration (ppm), the dye concentration at equilibrium (ppm), the initial MSW weight (g), and the solution volume (L) which was fixed (0.1 L) for all tests, respectively. Tests were carried out by mixing 0.25 g CMSW with 100 ppm MB and CR solutions at ambient temperature, constant pH (9.4), for 11 time points. Linearized and non-linearized Pseudo-first-order, second order, and the model of intra-particle diffusion were utilized as a part of the study to understand the adsorption mechanism of CMSW for CR and MB dyes and the applied kinetic modeling equations are calculated using eq3s to eq7s, respectively as shown below in (SI) [2- 6].

linear and Non-linear pseudo-first-order models

$$\log\ (Q_{e1} - Q_t) = \log Q_{e1} - \frac{K_1}{2.303}\,\mathbf{t} \qquad \textbf{Eq. S3}$$

linear pseudo-first-order form where Q_t**,** and K_1 are the amount of the adsorbed dyes at time t (ppm) and pseudo-first-order rate constant (min^{-1}), respectively. While Non-linear pseudo-first-order form is presented as following in **Eq. S4**

$$Q_t = Q_e \left(1 - e^{-K_1 t} \right)$$ **Eq. S4**

linear and Non-linear pseudo-second-order models

$$\frac{t}{Q_t} = \frac{1}{K_2 Q_{e2}^2} + \frac{t}{Q_{e2}}$$ **Eq. S5**

linear pseudo-second-order form where K_2 is the pseudo-second-order rate constant **(**g min/mg**).** While Non-linear pseudo-second-order form is presented as following in **Eq. S6**

$$Q_t = \frac{K_2 \, t \, Q_{e2}^2}{1 + K_2 \, t \, Q_{e2}^2}$$ **Eq. S6**

linear and Non-linear solving regression method for intra-particle diffusion model

$$Q_t = k_{id} \, t^{1/2} + C$$ **Eq. S7**

form where k_{id} is the intra-particle diffusion rate constant (mg $min^{1/2}$ g^{-1}) and C is a constant, respectively.

Text S1.2 Effect of pH

Since the pH of the solution impacts the surface charge of the adsorbent, therefore in order to find the ideal conditions for dye removal, the impact of pH on the adsorption mechanism was studied. Experiments were performed at initial pH values of 2, 4, 6, 8, 10, and 12 as adjusted with 0.1 M HCl and 0.1 M NaOH. Tests were carried out by mixing 0.50 g of the MSW with 100 ppm MB and CR solutions at ambient temperature and 120 min contact time.

Text S1.3 Effect of CMSW mass

Amounts of 0.10, 0.25, 0.50, 0.75, 1.00, 1.25, 1.50, 1.75, and 2.00 g of the MSW were tested with 250 ppm of MB and CR at ambient temperature and tested at 120 min contact time with pH = 12 for MB and pH = 2 for CR.

Text S1.4 Effect of initial dyes concentrations and isotherm models

Initial dyes concentrations of 10, 25, 50, 75, 100,125, 150, 175, 200, 225, and 250 ppm for both MB and CR were prepared with 0.10 g of MSW at ambient temperature and tested at 120 min contact time with pH = 12 for MB and pH = 2 for CR. The obtained data was fitted to the Langmuir and Freundlich isotherm models in their linear and non-linear regression forms [2-4], [7] in order to understand the interaction behavior between dyes and solid MSW adsorbent. Langmuir is predicated on the idea that dissolved dye is adsorbed in a monolayer form at predetermined homogeneous sites across the adsorbent. Linear, and nonlinear forms of Langmuir and Langmuir adsorption constant, R_L, or the separation factor which is a dimensionless equilibrium parameter are formulated in eq8s, eq9s, and eq10s respectively as given in SI. Values of R_L show the type of adsorption that can be anticipated. $R_L = 0$ indicates that the adsorption process is irreversible, and adsorbate cannot be removed; $0 < R_L < 1$ means that the adsorption process is a favorable process, while $R_L > 1$ means that the adsorption process is unfavorable [2]. Freundlich model describes heterogeneous multilayer adsorption as formulated in eq11s and eq12s as given in SI.

linear and Non-linear Langmuir isotherm equations

$$\frac{C_e}{Q_e} = \frac{1}{b\,Q_{max}} + \frac{C_e}{Q_{max}}$$ **Eq. S8**

linear form of Langmuir isotherm where b and Q_{max} are Langmuir constant (Lmg^{-1}) and the maximum amount of dye at complete monolayer coverage per unit mass of MSW (mmol/g), respectively. While Non-linear Langmuir isotherm form is presented as following in **Eq. S9**

$$Q_e = \frac{b\,C_e\,Q_{max}}{1 + b\,C_e\,Q_{max}}$$ **Eq. S9**

Langmuir adsorption constant (R_L)

$$R_L = \frac{1}{(1 + b * C_i)}$$ **Eq. S10**

linear and Non-linear Freundlich isotherm equations

$$\ln Q_e = \ln K_f + {}^{1}/_{n} \ln C_e$$ **Eq. S11**

where K_f and n are Freundlich constant that reveals the adsorption capacity (mg g^{-1}) and adsorption intensity, respectively. While Non-linear Freundlich isotherm form is presented as following in **Eq. S12**

$$Q_e = k_f \ C_e^{1/2} \qquad \textbf{Eq. S12}$$

Text S1.5 Effect of temperature and thermodynamic parameters

To better comprehend how temperature affects the adsorption of MB and CR dyes using the MSW adsorbent, tests were carried out on separate mixtures of 0.25 of MSW with 50 ppm MB and CR solutions, at 120 min contact time with pH = 12 for MB and pH = 2 for CR and tested at temperatures of 296, 308, 318 and 328K. Thermodynamic parameters [8, 9] examined are given in eq13s, eq14s, eq15s as shown in SI.

$$\Delta G^{\circ} = \Delta H^{\circ} - T\,\Delta S^{\circ} \qquad \textbf{Eq. S13}$$

$$\Delta G^{\circ} = - RT \ln K_d \ , \ K_d = \frac{Q_e \times m}{C_e \times v} \qquad \textbf{Eq. S14}$$

$$\ln K_d = \frac{-\ \Delta G^{\circ}}{RT} = \frac{-\ \Delta H^{\circ}}{RT} + \frac{\Delta S^{\circ}}{R} \qquad \textbf{Eq. S15}$$

where ΔG°, ΔH°, T, ΔS° , K_d , R are the change in Gibbs Free Energy (kJ/mol), enthalpy change (kJ/mol), Temperature in kelvin (K), entropy change (J/K mol), equilibrium dissociation constant, and gas constant (8.314 J/mol K), respectively. ΔG°, ΔH°, ΔS° values can be figured out from the $\ln K_d$ versus $^{1}/_{T}$.

Table S1: Parameters and Experimental conditions of MB and CR adsorption tests

Parameters	Values	Experimental conditions
Contact Time (min)	5,10,20, 40, 60, 80, 100, 120, 140, 160 and 180	T= 296 K, pH=9.4, 0.25 g CMSW, 100 ppm for MB and CR
pH	2, 4, 6, 8, 10, and 12	T= 296 K, 120 min, 0.50 g CMSW, 100 ppm for MB and CR
CMSW weight (g)	0.10, 0.25, 0.50, 0.75, 1.00, 1.25, 1.50, 1.75, and 2.00	T= 296 K, 120 min, 250 ppm for both dyes where MB at pH = 12 and CR at pH = 2
Initial Dyes concentrations (ppm)	10, 25, 50, 75, 100,125, 150, 175, 200, 225, and 250	T= 296 K, 120 min, 0.10 g for both dyes, pH = 12 for MB and pH = 2 for CR
Temperature (K)	296, 308, 318, and 328	120 min, 0.25 g CMSW, 50 ppm for MB and CR, pH = 12 for MB and pH = 2 for CR

Text S2: Experimental conditions for the Optimization of MB and CR adsorption capacities onto CMSW via CCD and RSM

Optimization of MB and CR adsorption capacities onto the MSW via CCD and RSM.

In this work, pH, temperature, the initial concentration of dyes, and MSW weight were treated as independent variables, while the adsorption capacities of dyes served as the dependent response variable with a fixed contact time of 120 min. The values of all parameters determined via the CCD approach are listed in Table S2 of SI, while the number of experiments determined by **eq16s as shown below in SI** [10]. Three vital steps comprise the optimization process, namely conducting statistically designed experiments, estimating coefficients in a statistical approach, and

predicting the response while verifying the adequacy of the model. The quadratic polynomial equation displayed in Eq **eq17s** as shown below in SI was employed to establish the empirical relationship between output response and the four independent variables [11].

$$N = 2^n + 2n + k_0 \qquad \textbf{Eq. S16}$$

Where, the value of n, which represents the number of independent variables, equals 4, and the number of central points, denoted by k_0, is 7.

$$Y = c_0 + \sum c_i x_i + \sum c_{ii} x_i^2 + \sum\sum c_{ij} x_i x_j \qquad \textbf{Eq. S17}$$

Where Y represents the calculated output response; x_i , x_j independent variables while c_0 , c_i , c_{ii} , c_{ij} are associated with constant, linear effect, quadratic effect, and linear interaction regression coefficients, respectively.

Table S2: Coded levels of the independent parameters used in the RSM.

Variables	symbols	Coded levels				
		-α	-1	0	+1	+α
Initial dye concentration (ppm)	X_1	50	100	150	200	250
CMSW weight (g)	X_2	0.1	0.60	1.10	1.60	2.1
Temperature (K)	X_3	296	304	312	320	328
pH	X_4	2	4.5	7	9.5	12

Text S3: A detailed description of constructing linear and nonlinear kinetics models.

The values of Q_{e1} and K_1 for both MB and CR were determined from the intercept and slope of $\log(Q_{e1} - Q_t)$ versus t, respectively as shown in Figure 3a, based on the linearized pseudo 1st order equation. A plot of $\frac{t}{Q_t}$ versus **t** in Figure 3c was derived from the linearized pseudo 2nd order model to determine the values of Q_{e2} and K_2 for both MB and CR from the slope and intercept, respectively. Figure 3e presents a graph of Q_t versus $t^{1/2}$ for linear regression analysis of intra-particle diffusion model used to obtain k_{id} and C for both MB and CR. For non-linear regression equations of pseudo 1st and 2nd order, solver function in Excel was used to find the best correlation values of (R^2) by manipulating Q_{e1} , Q_{e2} **,** $K_1$and K_2 as shown in Figure 3b, d of Q_t versus **t , while**

solver function applied to find the best correlation value (R^2) by manipulating k_{id} and C for both MB and CR for non-linear regression analysis of intra-particle diffusion model as shown in Figure 3f.

2. Results

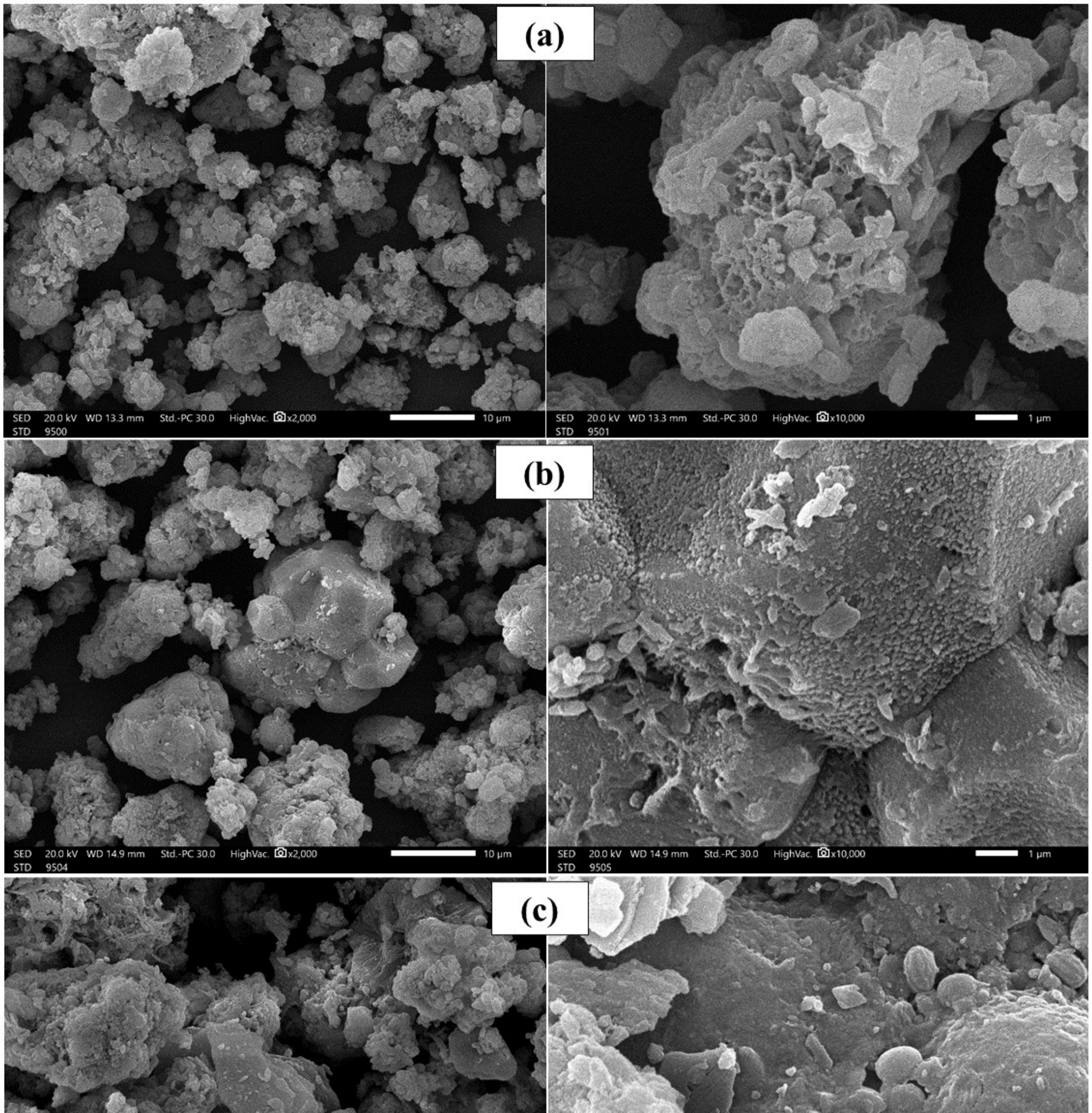

Figure S1: SEM images of CMSW before and after MB or CR adsorption where (a) pristine CMSW, (b) CMSW after MB adsorption and (c) CMSW after CR adsorption.

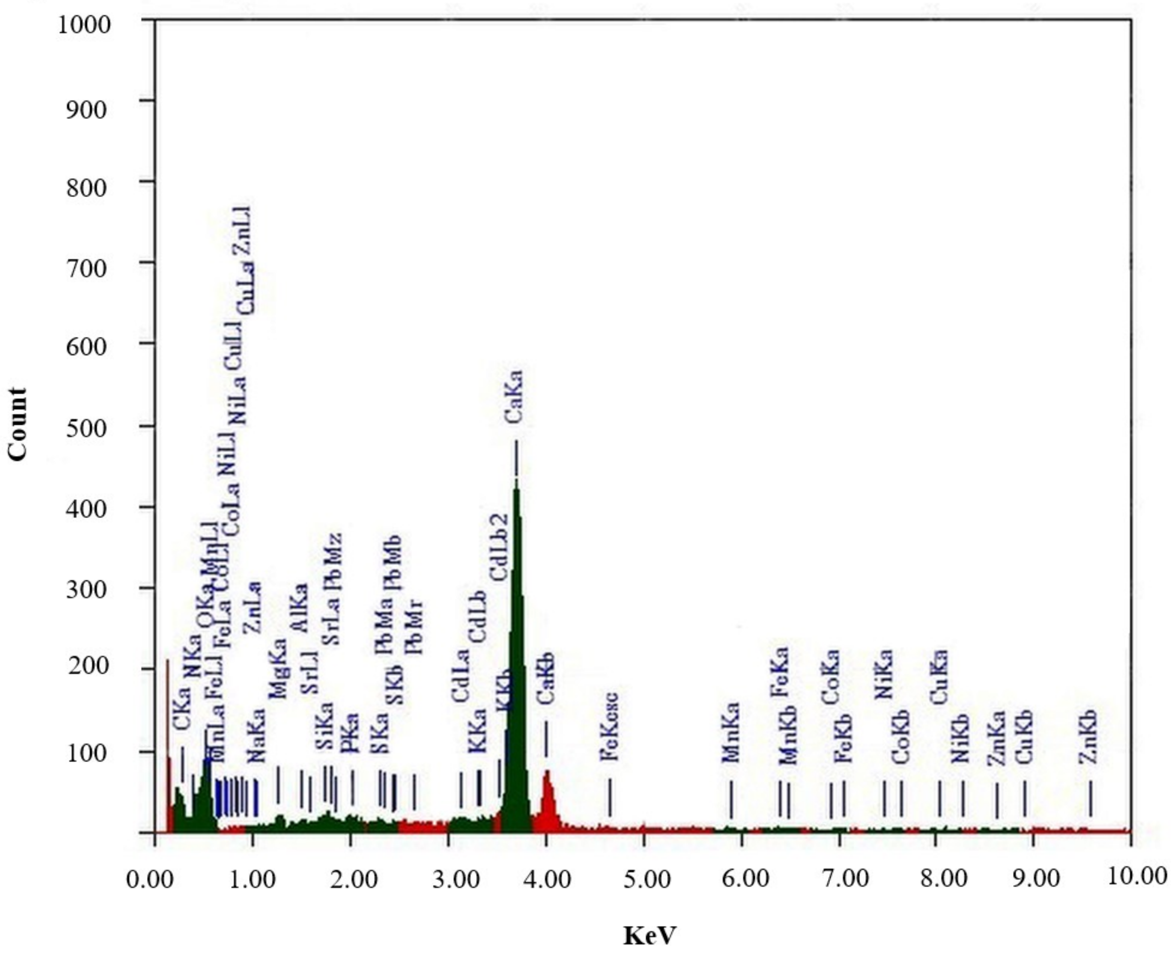

Figure S2**:** EDX spectra of elemental map analysis for CMSW

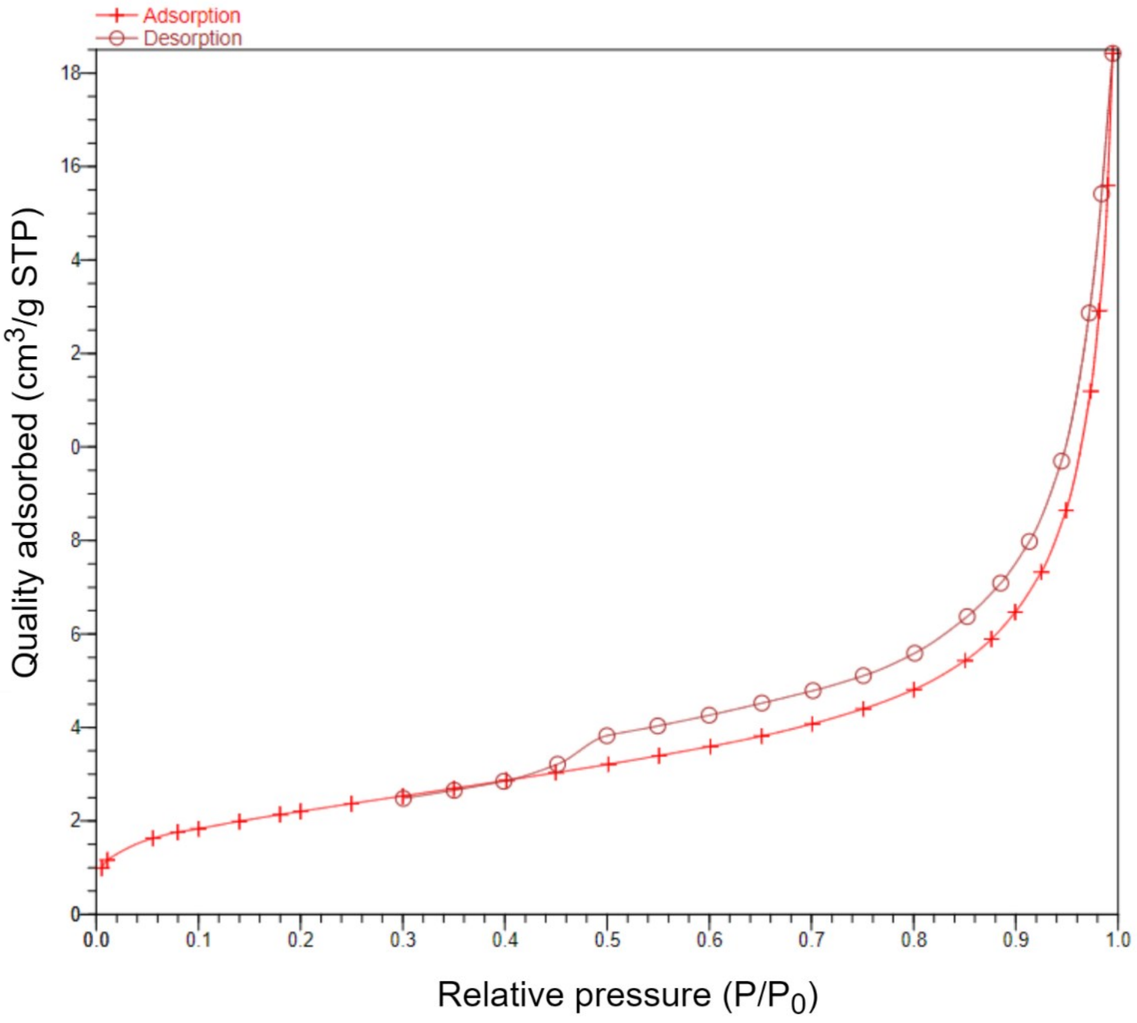

Figure S3**:** BET isotherm type II significant interaction involving macro porous adsorbent MSW.

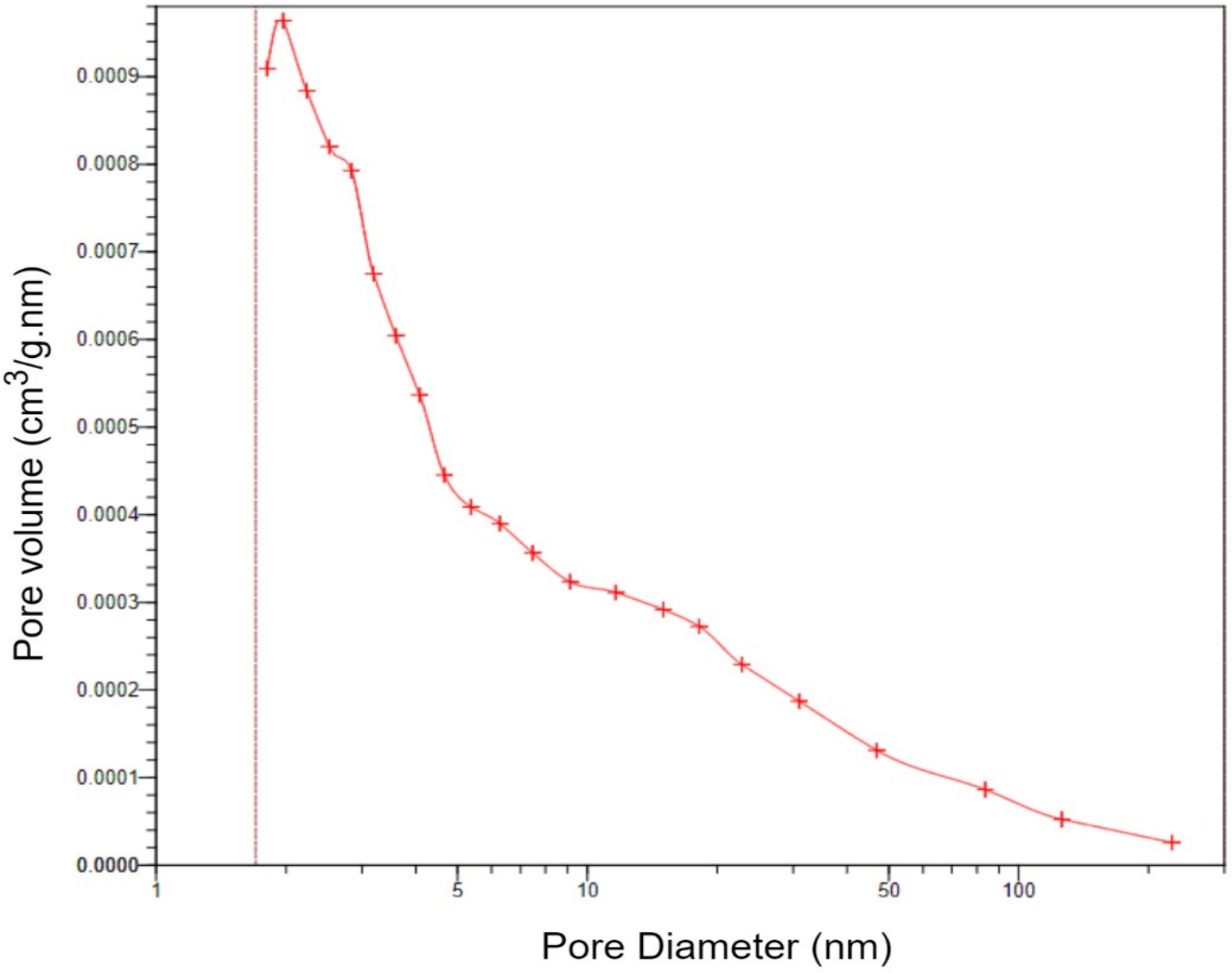

Figure S4: dispersion of the BET isotherm for pore size for the MSW.

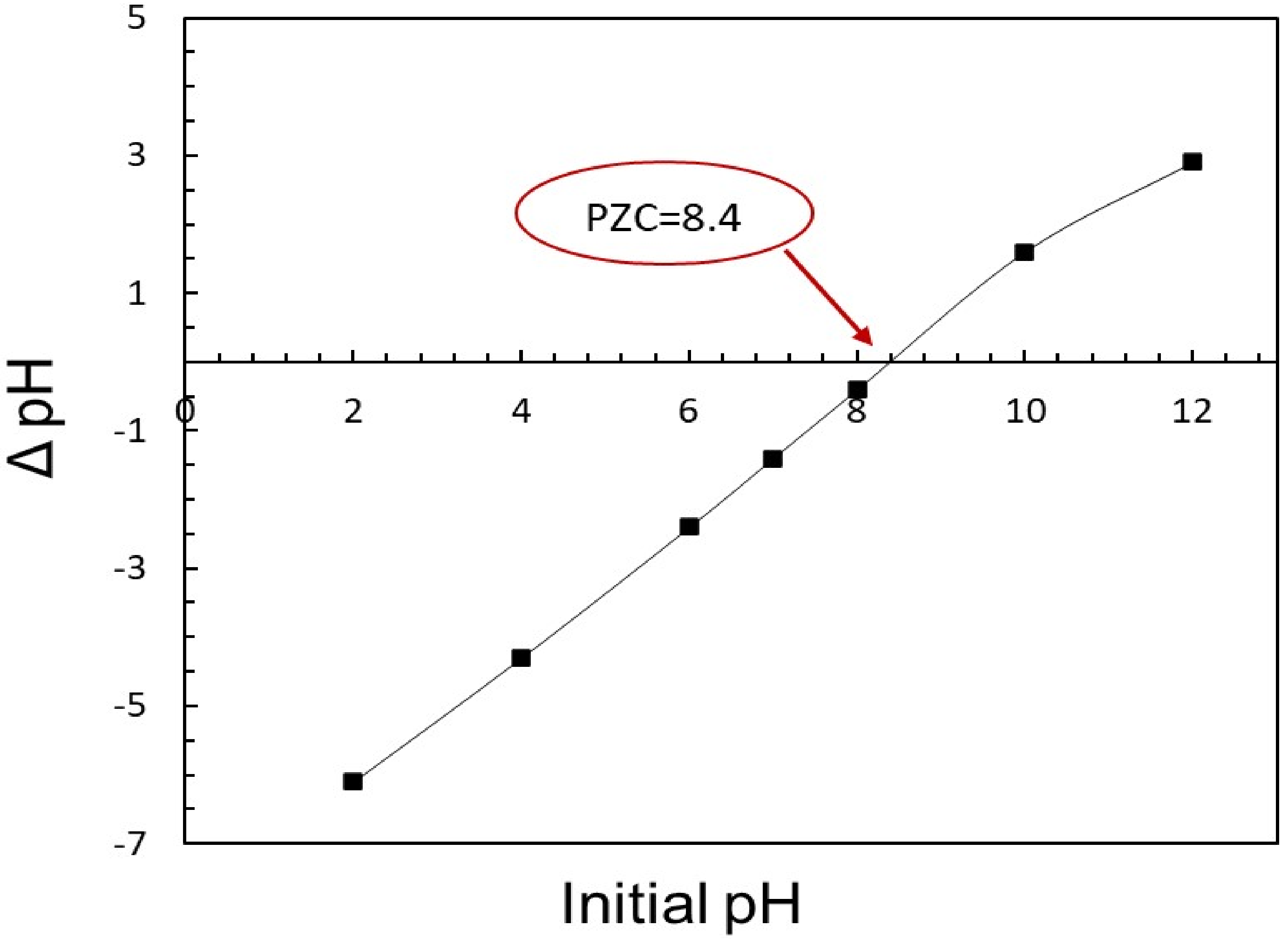

Figure S5. Point of zero charge (PZC) for the MSW from plot of Δ pH against initial pH.

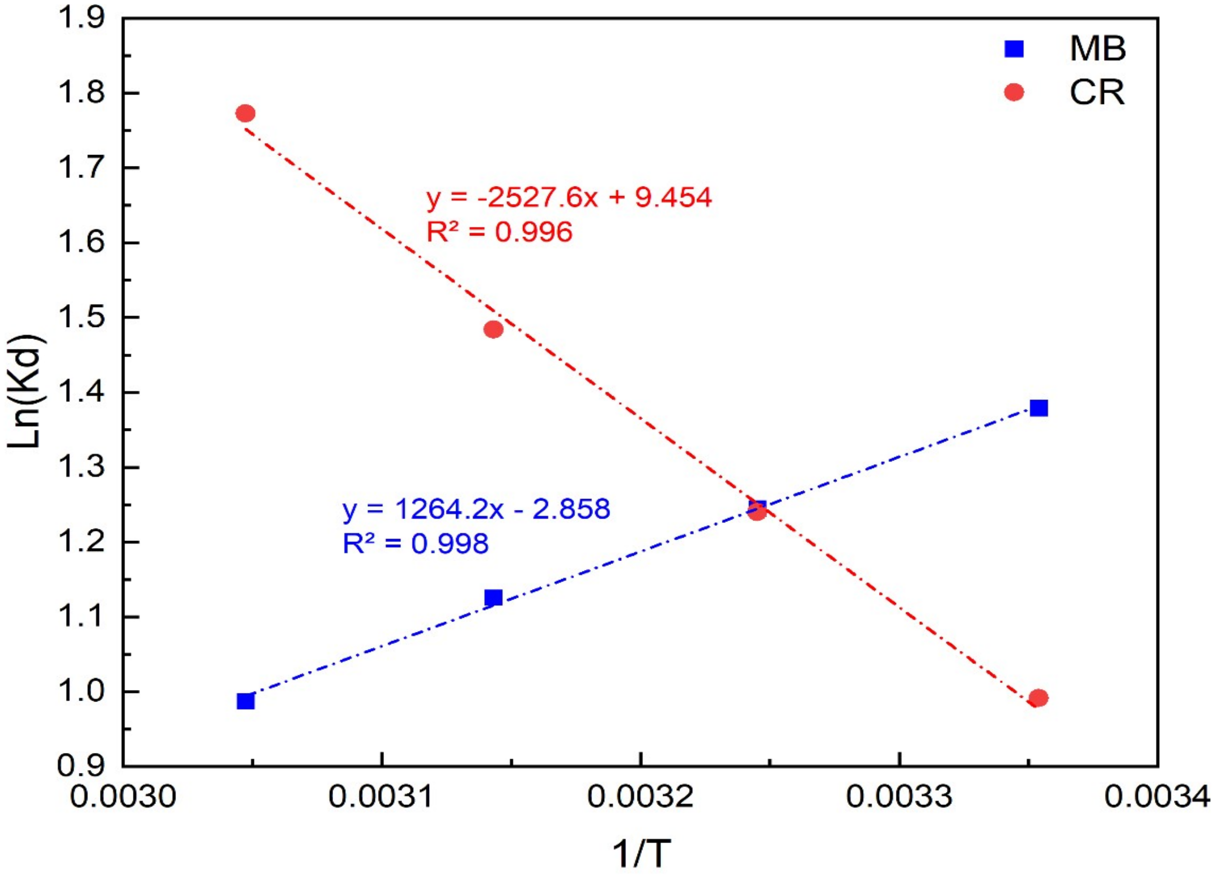

Figure S6: Thermodynamics for the adsorption of MB or CR onto the MSW.

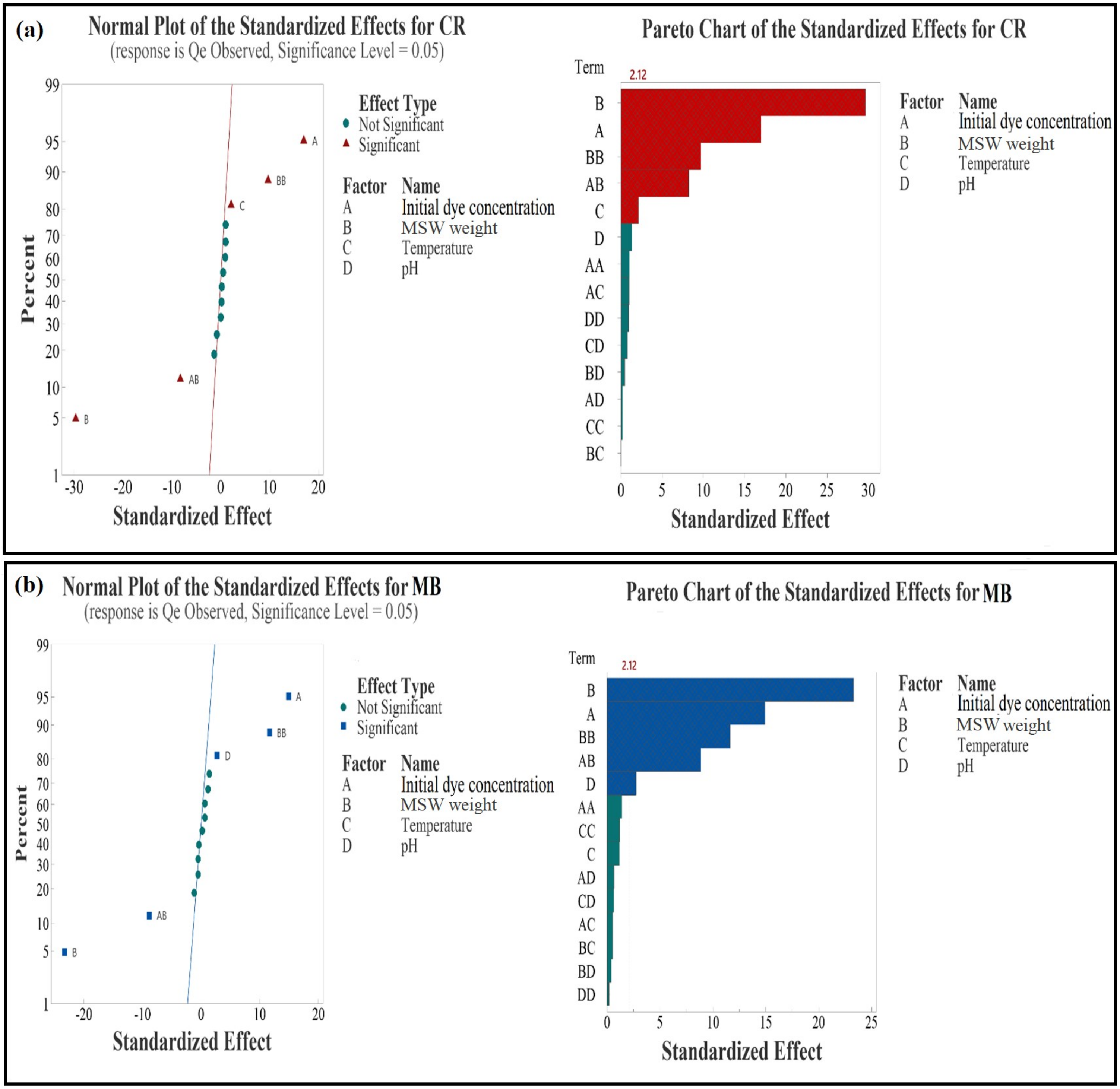

Figure S7. Significant and insignificant regression coefficients of CR model (Panel a) and MB Model (Panel b).

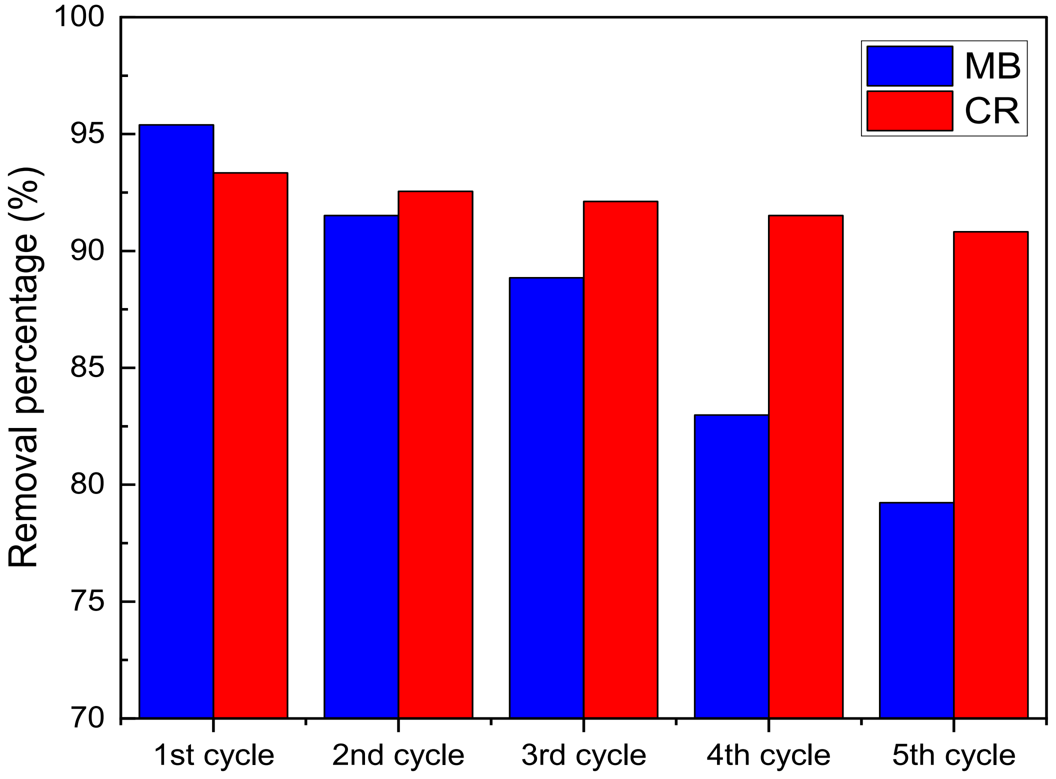

Figure S8: The removal percentage of MB and CR through adsorption onto MSW after five regeneration cycles. The data corresponds to the use of 0.25g of MSW at dye concentrations of 100 PPM and pH = 9.4 at room temperature

Table S3: Elemental map analysis of CMSW

Element	abbreviations	Normal Wt.%
Calcium	Ca	49.1
Oxygen	O	32.85
Carbon	C	11.19
Magnesium	Mg	1.45
Silicon	Si	0.83
Phosphorus	Pp	0.72
Aluminum	Al	0.48
Cooper	Cu	0.45
Iron	Fe	0.42
Sulfur	S	0.41
Strontium	Sr	0.38
Zine	Zn	0.38
Lead	Pb	0.33
Potassium	K	0.32
Cobalt	Co	0.32
Manganese	Mn	0.32
Sodium	Na	0.3
Cadmium	Cd	0.23
Total		100%

Table S4: Observed and predicted adsorption capacities of the CCD model for the CR and MB adsorption on the MSW.

Coded Factors					CR		MB	
					Q_e (adsorption capacity) mg/g			
Run Order	X1	X2	X3	X4	Exp	pred	Exp	pred
1	-1	1	-1	1	4.09	4.47	6.21	6.55
2	0	0	-2	0	10.30	9.94	13.15	12.74
3	-1	-1	-1	-1	13.62	14.03	12.11	13.48
4	1	-1	-1	1	24.57	25.78	31.17	32.26
5	1	-1	1	-1	27.03	28.14	29.03	28.88
6	-1	-1	-1	1	14.11	13.46	13.85	14.54
7	0	2	0	0	5.99	5.19	7.05	9.27
8	0	0	0	0	11.45	10.78	10.58	10.56
9	1	1	-1	1	7.08	7.30	10.99	10.13
10	-2	0	0	0	3.80	3.63	4.54	2.50
11	0	0	0	0	8.58	10.78	10.16	10.56
12	0	0	2	0	13.32	11.96	12.05	11.21
13	1	-1	1	1	27.02	26.91	30.99	32.00
14	-1	-1	1	1	12.14	13.43	13.55	15.13
15	2	0	0	0	21.26	19.71	21.14	21.92
16	0	0	0	0	10.95	10.78	10.58	10.56
17	-1	-1	1	-1	14.75	14.90	11.03	13.04
18	-1	1	-1	-1	4.02	4.50	5.95	6.09
19	1	-1	-1	-1	25.67	26.11	29.33	30.16
20	0	0	0	0	10.87	10.78	10.67	10.56
21	-1	1	1	1	4.53	4.45	5.99	6.31
22	0	0	0	-2	13.25	12.22	9.49	8.99
23	1	1	1	-1	8.12	9.14	6.08	6.53
24	-1	1	1	-1	5.11	5.39	5.72	4.82
25	0	0	0	0	11.55	10.78	10.64	10.56
26	0	0	0	0	10.93	10.78	10.66	10.56
27	1	1	1	1	7.36	8.45	10.22	9.05
28	1	1	-1	-1	6.89	7.09	10.02	8.64
29	0	0	0	2	11.65	10.96	13.32	12.57
30	0	0	0	0	10.97	10.78	10.51	10.56
31	0	-2	0	0	34.10	33.18	43.09	39.61

Exp: Experimental; pred: Predictive

Table S5: Predicted regression coefficients with SE, T-values, and P-values.

**Term**	**Coefficient**	**SE**	**T-Value**	**P-Value**	**Coefficient**	**SE**	**T-Value**	**P-Value**
	**CR**				**MB**			
**Constant**	10.757	0.437	24.62	0	10.543	0.603	17.48	0
x_1	4.012	0.236	17	**0 (S)**	4.859	0.326	14.92	**0 (S)**
x_2	-6.997	0.236	-29.65	**0 (S)**	-7.582	0.326	-23.28	**0 (S)**
x_3	0.502	0.236	2.13	**0.049 (S)**	-0.384	0.326	-1.18	0.255 **(IS)**
x_4	-0.313	0.236	-1.33	0.203 **(IS)**	0.89	0.326	2.73	**0.015 (S)**
x_1*x_1	0.222	0.216	1.03	0.32 **(IS)**	0.414	0.298	1.39	0.185 **(IS)**
x_2*x_2	2.101	0.216	9.72	**0 (S)**	3.471	0.298	11.63	**0 (S)**
x_3*x_3	0.042	0.216	0.19	0.848 **(IS)**	0.354	0.298	1.19	0.253 **(IS)**
x_4*x_4	0.202	0.216	0.93	0.364 **(IS)**	0.055	0.298	0.18	0.857 **(IS)**
x_1*x_2	-2.373	0.289	-8.21	**0 (S)**	-3.534	0.399	-8.86	**0 (S)**
x_1*x_3	0.289	0.289	1	0.332 **(IS)**	-0.21	0.399	-0.53	0.606 **(IS)**
x_1*x_4	0.059	0.289	0.21	0.84 **(IS)**	0.258	0.399	0.65	0.528 **(IS)**
x_2*x_3	0.004	0.289	0.02	0.988 **(IS)**	-0.206	0.399	-0.52	0.612 **(IS)**
x_2*x_4	0.134	0.289	0.46	0.648 **(IS)**	-0.151	0.399	-0.38	0.71 **(IS)**
x_3*x_4	-0.226	0.289	-0.78	0.446 **(IS)**	0.255	0.399	0.64	0.532 **(IS)**

S: Significant; IS: Insignificant

Table S6: ANOVA of CR and MB dye adsorption capacities

Source	DF	square of sequence	Contribution	Adj SS	Adj MS	F-Value	P-Value
**CR**							
**Model**	14	1789.52	98.82%	1789.52	127.82	95.66	0
**Linear**	4	1569.74	86.68%	1569.74	392.44	293.7	0
**Square**	4	127.17	7.02%	127.17	31.79	23.79	0
**2-Way Interaction**	6	92.61	5.11%	92.61	15.43	11.55	0
**Error**	16	21.38	1.18%	21.38	1.34		
**Lack-of-Fit**	10	15.41	0.85%	15.41	1.54	1.55	
**Pure Error**	6	5.97	0.33%	5.97	1		
**Total**	30	1810.9	100.00%				
**R-sq**	98.82%						
**R-sq(adj)**	97.79%						
**R-sq(pred)**	94.65%						
**MB**							
**Model**	14	2520.03	98.41%	2520.03	180	70.7	0
**Linear**	4	1968.8	76.88%	1968.8	492.2	193.33	0
**Square**	4	347.58	13.57%	347.58	86.9	34.13	0
**2-Way Interaction**	6	203.65	7.95%	203.65	33.94	13.33	0
**Error**	16	40.73	1.59%	40.73	2.55		
**Lack-of-Fit**	10	40.55	1.58%	40.55	4.05	128.21	
**Pure Error**	6	0.19	0.01%	0.19	0.03		
**Total**	30	2560.77	100.00%				
**R-sq**	98.41%						
**R-sq(adj)**	97.02%						
**R-sq(pred)**	90.87%						

Response Surface Methodology Equations

$$Q_{e\,(S\&IS)CR} = 10.757 + 4.012\,X_1 - 6.997\,X_2 + 0.502\,X_3 - 0.312\,X_4 \text{ [illegible]}$$
$$+\ 0.059\,X_1X_4 + 0.004X_2X_3 + 0.134X_2X_4 - 0.226\,X_3X_4 + 0.222\,X \text{ [illegible]}$$
$$0.202\,X_4^2$$

$$Q_{e\,(S\&IS)MB} = 17.5 + 0.2091\,X_1 - 21.65\,X_2 - 0.433\,X_3 - 0.44\,X_4 - \text{ [illegible]}$$
$$0.00206\,X_1X_4 - 0.0516X_2X_3 - 0.121X_2X_4 + 0.0128\,X_3X_4 + 0.0001 \text{ [illegible]}$$
$$0.0088\,X_4^2$$

$$Q_{e\,(S\&IS)CR} = 6.37 + 0.185\,X_1 - 17.88\,X_2 + 0.0628\,X_3 - 0.0949\,X_1 \text{ [illegible]}$$

$$Q_{e\,(S\&IS)MB} = 3.97 + 0.2527\,X_1 - 23.87\,X_2 + 0.356\,X_4 - 0.1413\,X_1 \text{ [illegible]}$$

References

[1] H. T. Nguyen, F. A. Ngwabebhoh, N. Saha, T. Saha, and P. Saha, “Gellan gum/bacterial cellulose hydrogel crosslinked with citric acid as an eco-friendly green adsorbent for safranin and crystal violet dye removal,” *Int J Biol Macromol*, vol. 222, pp. 77–89, 2022.

[2] A. M. Elgamal, N. A. Abd El-Ghany, and G. R. Saad, “Synthesis and characterization of hydrogel-based magnetite nanocomposite adsorbents for the potential removal of Acid Orange 10 dye and Cr (VI) ions from aqueous solution,” *Int J Biol Macromol*, vol. 227, pp. 27–44, 2023.

[3] R. R. Mohamed, M. H. Abu Elella, M. W. Sabaa, and G. R. Saad, “Synthesis of an efficient adsorbent hydrogel based on biodegradable polymers for removing crystal violet dye from aqueous solution,” *Cellulose*, vol. 25, pp. 6513–6529, 2018.

[4] F. A. Ngwabebhoh, M. Gazi, and A. A. Oladipo, “Adsorptive removal of multi-azo dye from aqueous phase using a semi-IPN superabsorbent chitosan-starch hydrogel,” *Chemical Engineering Research and Design*, vol. 112, pp. 274–288, 2016.

[5] H. Moussout, H. Ahlafi, M. Aazza, and H. Maghat, “Critical of linear and nonlinear equations of pseudo-first order and pseudo-second order kinetic models,” *Karbala International Journal of Modern Science*, vol. 4, no. 2, pp. 244–254, 2018.

[6] J. Wang and X. Guo, “Rethinking of the intraparticle diffusion adsorption kinetics model: Interpretation, solving methods and applications,” *Chemosphere*, vol. 309, p. 136732, 2022.

[7] R. Vitek and J. C. Masini, “Nonlinear regression for treating adsorption isotherm data to characterize new sorbents: Advantages over linearization demonstrated with simulated and experimental data,” *Heliyon*, vol. 9, no. 4, 2023.

[8] Z. Ren, X. Yang, W. Zhang, and Z. Zhao, “Preparation, characterization and performance of a novel magnetic Fe-Zn activated carbon for efficient removal of dyes from wastewater,” *J Mol Struct*, vol. 1274, p. 134407, 2023.

[9] H. K. Al-Hakeim, I. M. Al-Dahan, Z. H. Al-Hillawi, and R. S. Bustan, “Interaction of prolactin hormone with the surfaces of two new azo compounds,” *Int. J. Pharm. Pharm. Sci*, vol. 6, pp. 383–387, 2014.

[10] L. Soldatkina and M. Yanar, "Optimization of Adsorption Parameters for Removal of Cationic Dyes on Lignocellulosic Agricultural Waste Modified by Citric Acid: Central Composite Design," *ChemEngineering*, vol. 7, no. 1, p. 6, 2023.

[11] M. Jafari, M. R. Rahimi, A. Asfaram, M. Ghaedi, and H. Javadian, "Experimental design for the optimization of paraquat removal from aqueous media using a fixed-bed column packed with Pinus Eldarica stalks activated carbon," *Chemosphere*, vol. 291, p. 132670, 2022.
